# High-resolution high-throughput thermal neutron tomographic imaging of fossiliferous cave breccias from Sumatra

**DOI:** 10.1038/s41598-021-99290-0

**Published:** 2021-10-07

**Authors:** Holly E. Smith, Joseph J. Bevitt, Jahdi Zaim, Yan Rizal, Aswan, Mika Rizki Puspaningrum, Agus Trihascaryo, Gilbert J. Price, Gregory E. Webb, Julien Louys

**Affiliations:** 1 Australian Research Centre for Human Evolution, Environmental Futures Research Institute, Griffith University, Brisbane, QLD 4111 Australia; 2 Australian Centre for Neutron Scattering, Australian Nuclear Science and Technology Organisation, New Illawarra Rd, Lucas Heights, NSW 2234 Australia; 3 Geology Study Program, Institut Teknologi Bandung, Bandung, Jawa Barat 40132 Indonesia; 4 School of Earth and Environmental Sciences, The University of Queensland, Brisbane, QLD 4072 Australia

**Keywords:** Sedimentology, Palaeontology, Stratigraphy

## Abstract

We employ high-throughput thermal-neutron tomographic imaging to visualise internal diagnostic features of dense fossiliferous breccia from three Pleistocene cave localities in Sumatra, Indonesia. We demonstrate that these seemingly homogeneous breccias are an excellent source of data to aid in determining taphonomic and depositional histories of complex depositional sites such as tropical caves. X-ray Computed Tomographic (CT) imaging is gaining importance amongst palaeontologists as a non-destructive approach to studying fossil remains. Traditional methods of fossil preparation risk damage to the specimen and may destroy contextual evidence in the surrounding matrix. CT imaging can reveal the internal composition and structure of fossils contained within consolidated sediment/rock matrices prior to any destructive mechanical or chemical preparation. Neutron computed tomography (NCT) provides an alternative contrast to X-rays, and in some circumstances, is capable of discerning denser matrices impenetrable to or yielding no contrast with CT imaging. High-throughput neutron imaging reduces neutron fluence during scanning which means there is less residual neutron-induced radioactivation in geological samples; allowing for earlier subsequent analyses. However, this approach remains unutilised in palaeontology, archaeology or geological surveys. Results suggest that the primary agents in the formation of the breccias and concentration of incorporated vertebrate remains are several rapid depositional phases of water and sediment gravity flow. This study highlights the potential for future analyses of breccia deposits in palaeontological studies in caves around the world.

## Introduction

Tomographic imaging has been hailed as a new frontier in archaeology and palaeontology. Since the 1980s, the most practiced technique in these fields has been X-ray computed tomography (CT) (e.g. Tate et al.^1^, McGowan^2^, Macho et al.^3^, Stelzner et al.^4^, Smilg and Berger^5^). However, neutron tomography (NT) may be a more appropriate technique for palaeontologists studying fossil remains embedded in large volumes of rock or dense matrices, owing to the higher penetration of neutrons through these media (e.g. Schwarz et al.^6^, De Beer et al.^7^, Smith et al.^8^, Martins et al.^9^, Mays et al.^10^, Mayr et al.^11^). NT involves the transmission of neutral subatomic particles, neutrons, through an object, and the construction of a three-dimensional (3D) model that is computed from hundreds or thousands of resulting shadow radiographs acquired as the sample is rotated about its axis, for virtual analysis. Limited access to high-resolution NT facilities at nuclear research reactors and spallation neutron sources, combined with concerns regarding residual neutron-induced radioactivity of samples has inhibited broader application in palaeontology and archaeology. With recent improvements in technology and methods, the first palaeontological studies that use high-resolution NT techniques to inform on the formation and taphonomic histories of complex fossiliferous deposits at the macroscopic level have been published (e.g., Gee et al.^12^ and Louys et al.^13^).

In Southeast Asia, archaeologists and palaeontologists focus a great deal of their research on caves, as these systems are natural sites for the accumulation of sediment and faunal remains^13, 14^. Most of these remains are preserved in dense calcareous clastic rocks, called breccia, cemented to the walls, or accumulated on the floor. Karst breccias are commonly preserved as thin remnants of much larger, older deposits removed from cave networks during speleogenesis. Speleothem and mammalian teeth are commonly incorporated into these indurated deposits, and direct dating of these aggregates can provide a chronological framework of depositional history. However, the complicated depositional histories of breccia can lead to temporal mixing of incorporated aggregates^14–15^. Thus, the complex histories of karstic breccia deposits have the potential to confound the calculated ages and contemporaneity of faunal remains contained within them^14^. This can lead to inaccurate interpretations of conventional direct dating techniques when establishing the age of the deposits, as the fossils found within a single stratum may not be contemporaneous (e.g. Louys et al.^13^, O’Connor et al.^16^, Curnoe et al.^17^).

Hominid and associated faunal skeletal remains commonly bear indication of past processes and activities that are useful for taphonomic interpretation. Understanding the taphonomic processes that have acted upon a fossil assemblage, and to what degree, may have serious implications for interpretations of the significance of a fossil deposit and potential biases in a deposit or sample. Depositional processes not only modify bones but can dramatically alter the composition of entire assemblages, thus distorting demographic reconstructions^18, 19^. Therefore, research into the modes of accumulation and concentration are important in understanding taphonomic biases. However, the taphonomic characteristics of vertebrate-bearing deposits in the cave systems of Southeast Asia have commonly been poorly documented, and breccia are a particular problem. Detailed recording of spatial distribution data in such breccias in relation to dating samples may improve the reconstruction of pre- and post-depositional actions in vertebrate-bearing deposits. Current spatial distribution data collected from cave breccias in Southeast Asia are commonly limited to two-dimensional assessments and restricted to the surface of the deposit in the field, thus limiting interpretation and understanding of local taphonomic biases. However, the formation processes of a breccia can be studied in more detail to understand the mechanisms of faunal accumulations in such complex environments.

Information on the internal composition of karst breccia is commonly limited as any possible contextual information was often largely destroyed in the extraction of incorporated aggregates or was disregarded. Recently, however, cave breccia remnants cemented to the cave walls, floors and ceilings have been considered valuable archives for studying bio/chronostratigraphic sequences over long timespans, cultural evidence of human occupation and palaeoenvironmental change in the Quaternary (e.g. Westaway et al.^20^, Louys et al.^13^, O’Connor et al.^16^, Wiersma et al.^21^, Herries et al.^22, 23^). Therefore, a comprehensive study of breccia formation is needed to better understand faunal, archaeological and palaeoenvironmental histories of complex depositional sites such as tropical caves. Three-dimensional analyses of spatial distribution data using reconstructed volume data can be analysed to assess the spatial location, orientation and temporal sequence of sediment layers and clasts within a breccia, thus revealing significant evidence of depositional history and palaeoenvironment at the study site. Spatial associations of faunal remains may provide preliminary evidence of the agents of concentration that have influenced faunal assemblages incorporated into the breccia.

In a preliminary attempt to address these deposits, the first neutron tomographic imaging of fossiliferous breccia from a tropical cave in Southeast Asia was performed by Louys et al.^13^. A single breccia subsample from Matja Kuru TD, Timor-Leste was scanned using NT. Two grey-level volume rendered images were coloured and filtered to highlight different clasts. These data revealed evidence of overall breccia composition and the presence of certain textures that could be compared to nearby younger unconsolidated deposits. That study did not, however, create a detailed chronological resolution of formation history. A more comprehensive tomographic study of multiple samples within individual cave sites, and across multiple caves, may offer a more holistic insight into complex cave depositional environments. Here, we use the DINGO thermal-neutron tomography imaging station at the Australian Nuclear Science and Technology Organisation (ANSTO), Sydney, Australia^24^ to obtain a series of neutron-transmission radiographic images of thirteen breccia subsamples from Lida Ajer, Ngalau Gupin and Ngalau Sampit, caves in the Padang Highlands of Sumatra, Indonesia. The scans provide insights into the relative chronology of site formation and evidence of taphonomic agents impacting site formation.

### Study sites

**Figure 1:**
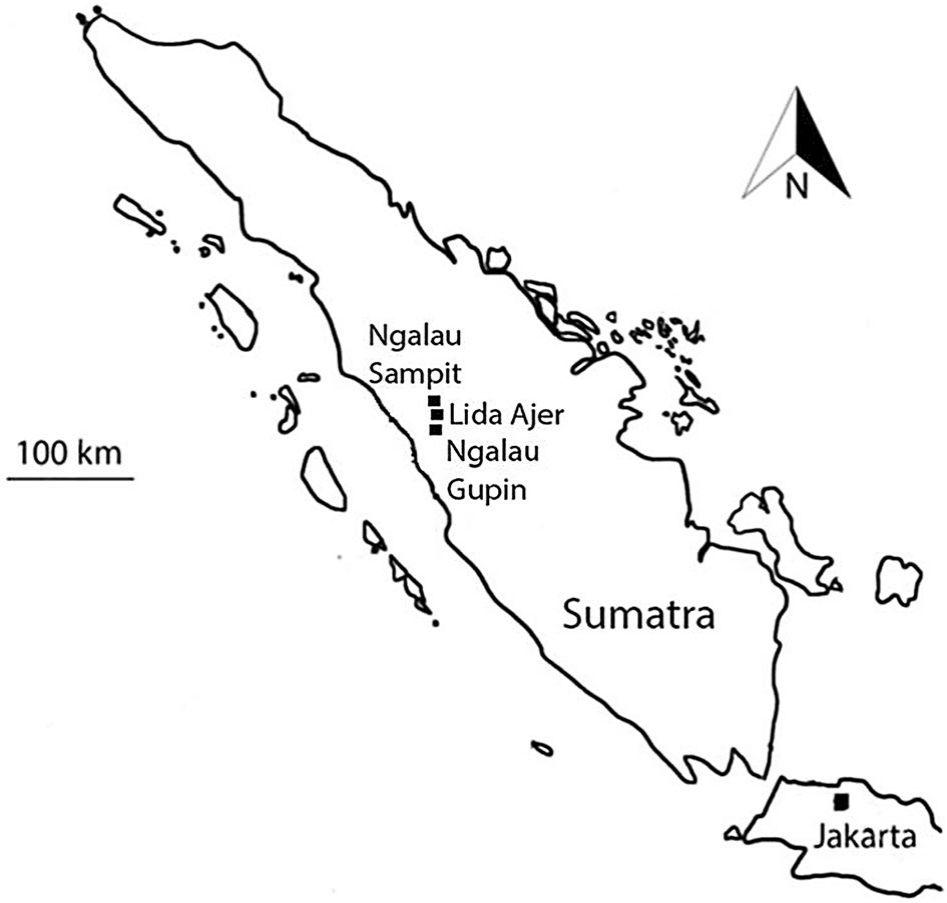
Locality map of the three cave sites in the Padang Highlands, Sumatra.

During a survey of caves in the Padang Highlands of western Sumatra, three key localities, Lida Ajer, Ngalau Gupin and Ngalau Sampit, were chosen for analysis and excavation (Fig. 1). These sites were chosen due to a considerable presence of fossil-bearing breccia deposits. They are detailed below.

### Lida Ajer

Lida Ajer, situated south of Payakumbuh, West Sumatra, is a large three-chambered cave complex initially surveyed by Eugene Dubois^25^ and later re-analysed by Westaway et al.^26^ and Louys et al.^13^. The main fossil-bearing chamber of the cave has four discrete breccia sites that have been reconstructed as the remnants of a single, chamber filling deposit. These breccias consist of consolidated high-density speleothem matrix with angular allogenic clasts. They are commonly fossiliferous, with most of the faunal material composed of tooth crowns. Westaway et al.^26^ recorded modelled ages of ~ 73–63 ka for deposition of the breccia in Lida Ajer, constrained by the ^230^Th age of a basal flowstone of 203 ± 17kyr (LA-F3) and a straw stalactite dated to 84 ± 1 ka.

### Ngalau Gupin

Ngalau Gupin is a single, large, cavernous chamber previously surveyed by Louys et al.^13^. This cave has two discrete breccias cemented onto the walls of the southernmost U-shaped passageway: one non-fossiliferous deposit on the eastern side of the western passage and one fossiliferous deposit in the western side of the western passage at the same topographic level. These breccias are consolidated calcareous deposits of high-density speleothem matrix with angular allogenic clasts. A small quantity of breccia residue and erosive scarring along the entirety of the sub-chamber walls indicates that this deposit was once much more extensive. Numerous tooth crowns occur on the breccia exterior.

### Ngalau Sampit

The third cave locality is Ngalau Sampit, a two-chambered complex accessed through a series of very narrow tunnels. This cave was partially surveyed by Louys et al.^13^, as this site consists of a main northern chamber above water level and a submerged southern chamber. The northernmost region of the northern chamber splits into an east and west bearing passageway. There are four remnant breccia deposits cemented to both east and west passageway ceilings: one at each entrance and termination. These breccias consist of consolidated high-density speleothem matrix with angular allogenic clasts. Louys et al.^13^ recorded that the fossils in the breccia deposits consist mostly of isolated mammal teeth.

## Methodology

### Excavation

A single block was extracted from each discrete consolidated breccia site in the three key cave localities using a hammer and chisel. Each breccia sample measured a maximum of fifteen centimetres in width or diameter, owing to the practical thermal-neutron transmission limits through similar calcareous specimens based on previous experience at the DINGO neutron tomography imaging station^13^. Breccia samples were extracted from the most heavily consolidated sub-section of each deposit to maximise preservation potential. Thirteen consolidated breccia deposits were extracted in total, each with a unique number (Table 1). The location of the breccia samples was noted onto the cave plans in the field. A north arrow and an ‘up’ arrow were drawn directly onto the samples to track the orientation of the internal geometry in relation to the overall sample in the cave site.

**Table 1:** Sample ID, site and locality of the thirteen consolidated breccia blocks.

Sample ID	Site	Locality
Reconsum18-53	Lida Ajer	LA-1
Reconsum18-52	Lida Ajer	LA-2A
Reconsum18-50	Lida Ajer	LA-3
Reconsum18-46	Lida Ajer	LA-4 above the flowstone
Reconsum18-47	Lida Ajer	LA-4 below the flowstone
Reconsum18-73	Ngalau Gupin	NG-A floor
Reconsum18-49	Ngalau Gupin	NG-A wall
Reconsum18-55	Ngalau Gupin	BC-2A
Reconsum18-71	Ngalau Sampit	BC-1
Reconsum18-54	Ngalau Sampit	BC-2
Reconsum18-48	Ngalau Sampit	BC-3
Reconsum18-72	Ngalau Sampit	BC-4
Reconsum18-51	Ngalau Sampit	SP-1

### Lida Ajer

**Figure 2:**
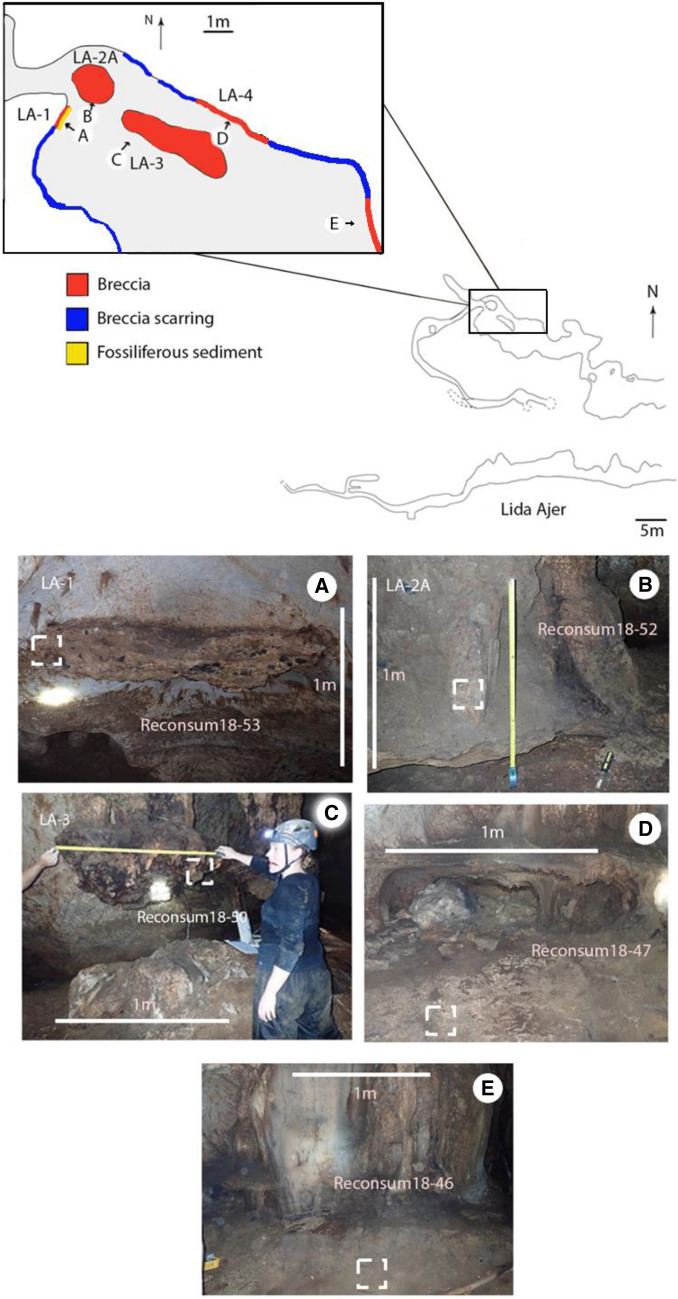
A scale profile map of Lida Ajer (above) and a scale plan map of Lida Ajer (inset) with a colour scale section highlighting the key breccia and fossiliferous sediment sites. (below) (**A**–**E**): Photographs of the key breccia sites in Lida Ajer, the white dashed box highlighting the exact extraction point of the samples. Arrows indicate the orientation of photographs A-E in relation to the inset scale profile map. (**C**) Co-author performing fieldwork.

Five samples in total were taken from four discrete breccia sites in Lida Ajer (Fig. 2). A single sample was taken from LA-1, LA-2 and LA-3, whereas two samples were taken from LA-4: one from above the flowstone deposit and one from below.

### Ngalau Gupin

**Figure 3:**
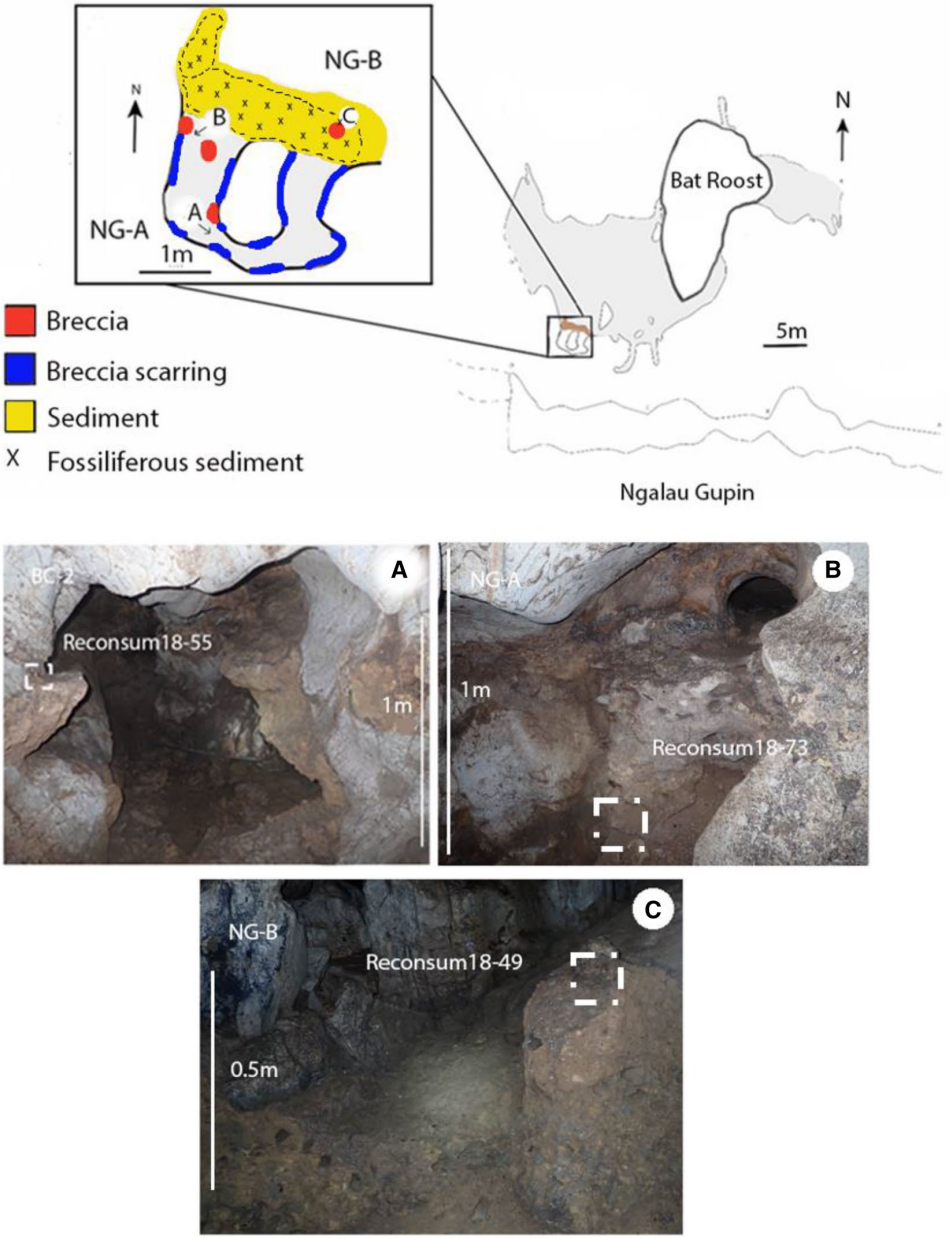
Plan view map of Ngalau Gupin (above) and a scale plan map of Ngalau Gupin (inset) with a colour scale profile section highlighting the key breccia and fossiliferous sediment sites (below) (**A**–**C**): Photographs of the key breccia sites in Ngalau Gupin, the white dashed box highlighting the exact extraction point of the samples. Arrows indicate the orientation of photographs (**A**–**C**) in relation to the cave plan.

Three samples in total were taken from three discrete breccia sites in Ngalau Gupin cave (Fig. 3). A single sample was taken from the non-fossiliferous BC-2A breccia. A sample was taken from the well-lithified wall section and from the poorly consolidated floor section of the NG-A breccia.

### Ngalau Sampit

**Figure 4:**
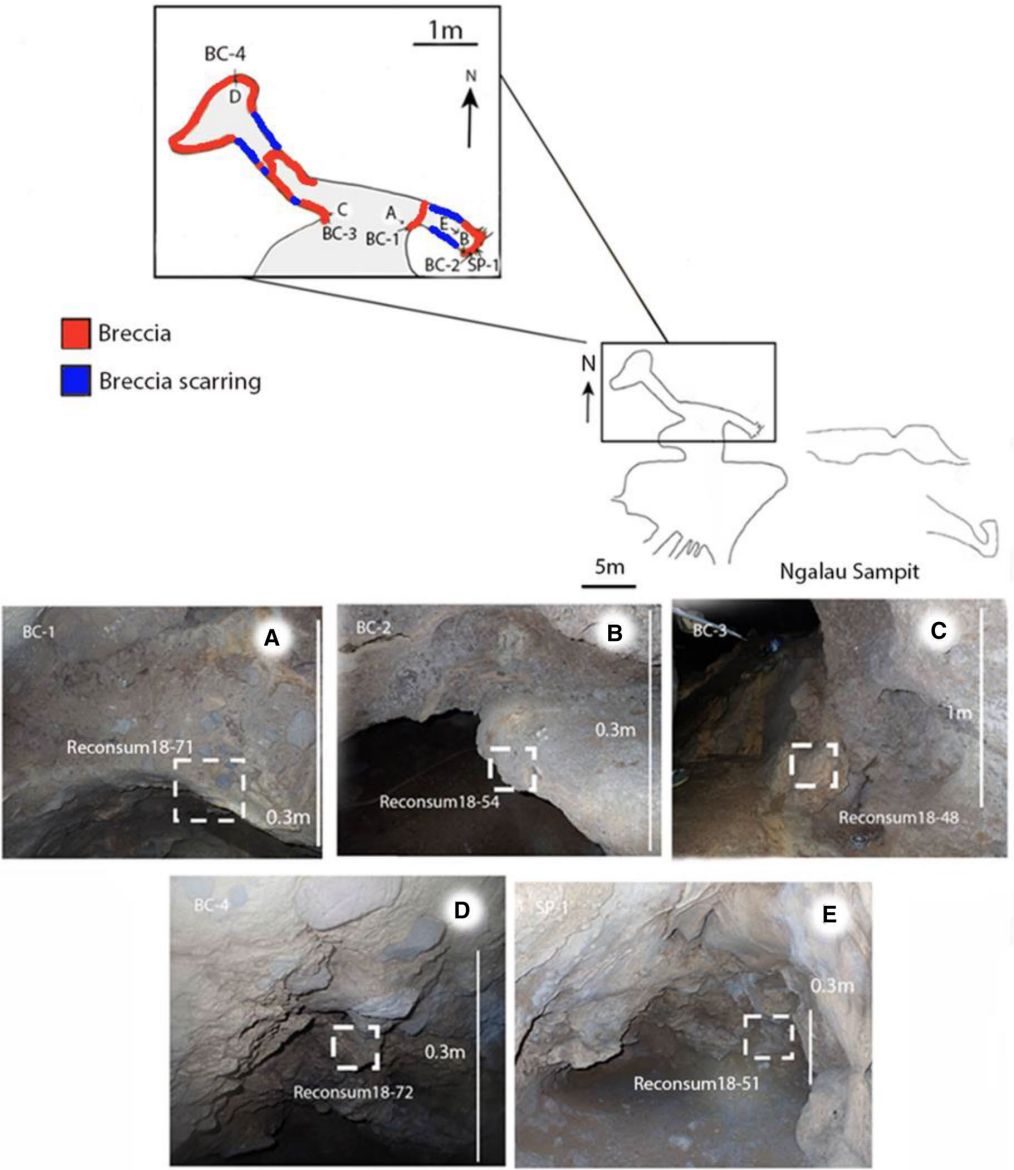
Scale profile map of Ngalau Sampit (above) and a scale plan map of Ngalau Sampit (inset) with a colour scale section highlighting the key breccia and fossiliferous sediment sites (below) (**A**–**E**). Photographs of the key breccia sites in Ngalau Sampit, the white dashed box highlighting the exact extraction point of the block samples. Arrows indicate the orientation of photographs (**A**–**E**) in relation to the cave plan.

Five samples in total were taken from five discrete breccia sites in Ngalau Sampit cave (Fig. 4). A single sample was taken from breccia remnants in each passage entrance (BC-1 and SP-1) and passage termination (BC-2 and BC-4). A single final sample was taken from the cave floor of the northern chamber at a slightly higher elevation than the passageways (BC-3).

### Drying of samples

The first, 10–24 h-long high-resolution NT scans of the breccia samples produced no, or substandard, results as the moisture content was extremely high. There is a high attenuation of neutrons by moisture content that significantly reduces the information content in the scan data. Thus, prior to the repeated neutron scans the samples were placed in an oven set at 50 °C for 3 h. The temperature was then raised to 90 °C at a ramp rate of 20 °C/h and the samples were maintained at that temperature for 8 h. The oven temperature was then decreased to room temperature at a ramp rate of 20 °C/h, and the samples placed in sealed bags until the start of each neutron imaging measurement to prevent moisture uptake.

### Neutron tomography

Attempts to conduct high-resolution synchrotron X-ray CT analysis of these breccia samples using the Imaging and Medical Beamline (IMBL) at the Australian Synchrotron, Melbourne, Australia, using a monochromatic beam energy of 110 keV, and a pink beam with peak energy of 260 keV were unsuccessful due to the high X-ray attenuation of these samples. Thermal-neutron tomographic measurements were performed at the DINGO thermal-neutron radiography/tomography/imaging station^24^ at ANSTO’s 20 MW OPAL nuclear research reactor, Sydney, Australia.

The DINGO instrument has two basic configurations; low-intensity mode with a collimation ratio (*L*/*D,* where *L* is the neutron aperture-to-sample length and *D* is the neutron aperture diameter) of 1000 and neutron flux of 1.15 × 10^7^ n cm^−2^ s^−1^ at the sample position, and a higher-intensity mode with a *L*/*D* of 500 and neutron flux of 4.75 × 10^7^ n cm^−2^ s^−1^ at the sample position. For this study, DINGO was configured in high-intensity mode and coupled with a Photometrics Iris 15™ Large Field of View sCMOS camera (5056 × 2968 pixel, 16-bit, 4.25 × 4.25 µm pixel area), a Zeiss Ikon 50 mm f/2.0 Makro Planar lens and 200 × 200 mm × 100 μm thick ZnS(Ag)/^6^LiF scintillator screen (RC Tritec AG) to achieve a Field-of-View of 115 × 197 mm with 38.9 × 38.9 μm pixels.

A total of 780 equally spaced angle shadow-radiographs of 2 s exposure each were obtained every 0.23° as each sample was continuously rotated 180° about their vertical axis. Twenty dark (closed shutter) and beam profile (open shutter) images were obtained for calibration both before and after shadow-radiograph acquisition. Total scan time for each specimen was 30 min. The radiographic data were binned by a factor of 2 using bin averaging to improve signal-to-noise, and achieve an effective voxel of 77.8 × 77.8 × 77.8 μm^3^. Tomographic reconstruction of the 16-bit raw data was accomplished using Octopus Reconstruction v.8.8^27^. Beam-hardening corrections were applied when necessary.

A great benefit of high-throughput neutron imaging is that it reduces the neutron fluence (the total neutrons striking sample) during scanning. As a small portion of penetrating neutrons interact directly with atomic nuclei in materials to formation radioactive isotopes, reduced scan times result in less residual radioactivation such that the accumulated residual activity of the breccia samples was so low that it decayed to natural levels within 3 days. The reduction in measurement time to 30 min per sample also enabled a high-throughput survey of breccia samples. Moisture was the greatest challenge in this research, and the deposits were completely obscured in the first round of neutron tomographic measurements. Furthermore, there were attenuation issues with clays and water after drying. X-rays interact with the cloud of electrons surrounding each atom, and thus are more highly attenuated by atoms of greater atomic number, and by higher density materials. Neutron scattering results from two fundamental interactions with atoms; the residual strong interaction, or nuclear force with atomic nuclei, and the electromagnetic interaction of the neutron’s magnetic moment with internal magnetic fields associated with unpaired electrons in atomic shells^28^. A consequence of these interactions is that the degree of neutron scattering and thus attenuation does not depend on atomic number, appearing to vary randomly across the periodic table, and significantly for different isotopes. This pattern of isotope sensitivity formed by the interaction of X-rays and neutrons with matter is well documented (e.g. Vontobel et al.^29^, Figs. 3 and 4, Tengattini et al.^30^, Fig. 1). The elemental, and isotopic abundance in the samples define the neutron-attenuation (scattering plus absorption) properties of the rock and the apparent densities of the clasts and matrix, with neutron-scattering by hydrogen being the dominant attenuation factor in these specimens. It is critical throughout the paper not to confuse actual density with neutron-apparent density.

### Visualisation

VGStudio Max 3.4 (Volume Graphics GmbH) was used to render the resulting stacks of two-dimensional digital slices into three-dimensional images for virtual reconstruction of the subsamples and allocation of sectioning planes. These three-dimensional images were analysed with the purpose of providing geometric evidence, such as volume, area, orientation, and direction of individual clasts incorporated within the breccia sample on the sectioning planes. Additionally, the matrix was visualised to identify any sedimentological structures and depositional features indicative of chronological and/or taphonomic history. The quality of the images was enhanced by variable changes of contrast, grey levels, sharpening and brightening with VGStudio image processing tools. The dimension of the relevant morphological features in the images are ~ 0.5 mm, or greater.

### Analysis

**Figure 5:**
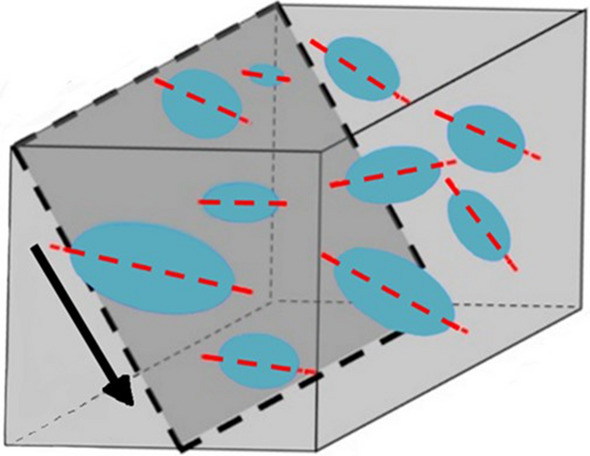
A representative figure to show slope and slope direction as described in the manuscript. The grey cube represents a sediment matrix, and the blue ovals represent individual clasts ‘floating’ within. The plane outlined by the dashed lines is the incline and the black arrow shows the slope direction and amount. Red dashed lines represent the steepest angle, or ‘slope direction’ of the clasts relative to a horizontal plane—herein 20° to 70° east.

**Figure 6:**
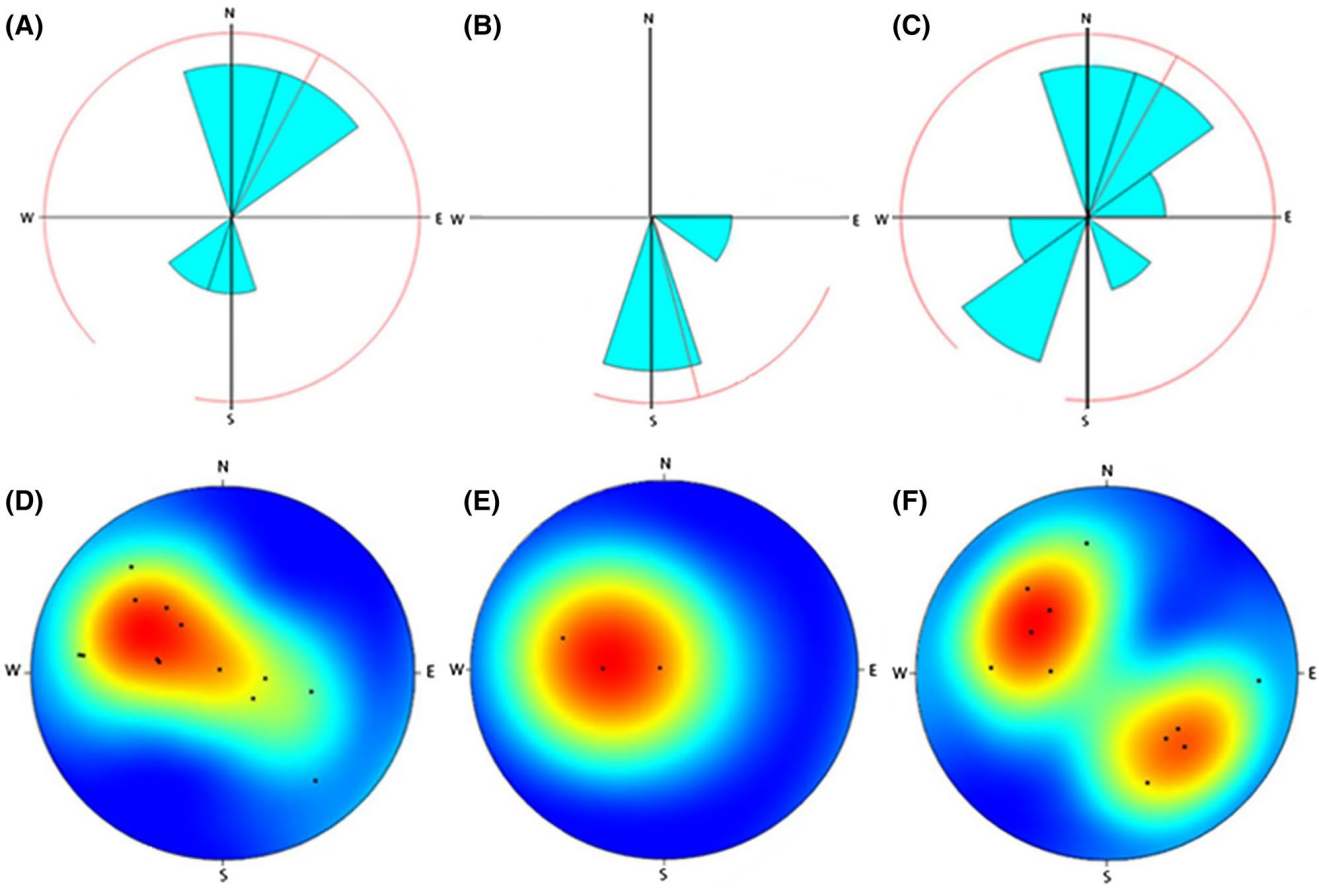
(**A**–**C**) Orientations of internal clasts of the discrete breccia subsamples in relation to the cave passage orientations from: (**A**) Lida Ajer cave (5 subsamples); (**B**) Ngalau Gupin cave (3 subsamples); (**C**) Ngalau Sampit cave (4 subsamples). Figures (**A**–**C**) are calculated using geometrical directions (one sample) function^36^. The red lines denote the mean angle. (**D**–**F**) Stereoplots of the slope and slope direction of the internal clasts of the breccia subsamples in relation to the cave passage orientations and slopes from: (**D**) Lida Ajer cave; (**E**) Ngalau Gupin cave; (**F**) Ngalau Sampit cave. Figures (**D**–**F**) are calculated using a spherical function^36^. Colour warmth maps the commonality of clasts sloping in one direction, with blue denoting no or few clasts to red denoting the greatest number of clasts.

The grain sizes in the subsamples were classified following Udden^31^, Wentworth^32^, and Blair and McPherson^33^. The lithology of the breccia was determined following Stow^34^ and Tucker^35^, dividing the principal breccia types into extra-formational, intra-formational and cataclastic, and deriving the corresponding information on the nature and origin of the deposit. The relationships between the clasts in each breccia deposit are quantified by tabulating each void and clast and noting maximum lengths and widths, orientation and shape. As the orientation data for each deposit is recorded directly onto the sample at the point of excavation, the slope and slope direction can be mapped directly on the scale cave profile. Analysing these data has the potential to reveal the origin, transport mechanism, depositional history, and stratigraphy of the breccia. In turn, this gives critical insights into the taphonomic history of the faunal remains cemented within. The full table is provided in Supplementary Information. The percentage of clasts in each discrete breccia sample of a particular shape, and the average result for each individual cave, with the average result are shown in Table 2. The mean maximum length and width, and the slope and slope direction of the internal clasts of the thirteen discrete breccia subsamples are shown in Table 3. PAST 2.17c^36^ was used to graphically represent the slope and slope direction of the internal clasts of each discrete subsample from the three key cave sites. The orientations of the internal clasts are depicted using the geometrical directions (one sample) function. The slope of the clasts describes the attitude relative to a horizontal plane and slope direction of the clasts describes the steepest angle of descent relative to a horizontal plane (Fig. 5). The slope directions of the internal clasts are depicted as stereoplots using the spherical function (Fig. 6).

**Table 2:** The percentage of clasts in each discrete breccia sample of a particular shape, and the average result for each individual cave, with the average result calculated below.

Cave site	Sub sample	Quadrant	Rounded	Equant	Irregular	Tabular	Elongate	Discoid	Globular	Cubic	Prismatic	Total number of clasts
Lida Ajer	Reconsum18-53	0	62	12	0	0	10	16	0	0	0	49
	Reconsum18-52	0.01	98	0	0.3	0	0.69	1	0	0	0	614
	Reconsum18-50	0	75	6	7	5	2	5	0	0	0	291
	Reconsum18-47	0	99.5	0	0.3	0	0.1	0.1	0	0	0	664
	Reconsum18-46	0	60	0	13	0	27	0	0	0	0	15
	Cave average	0	78.9	3.6	4.1	1	8	4.4	0	0	0	
Ngalau Gupin	Reconsum18-73	0	100	0	0	0	0	0	0	0	0	17
	Reconsum18-55	0	0	0	0	0	0	0	0	0	0	0
	Reconsum18-49	0	33	0	66	0	0	0	0	0	0	3
	Cave average	0	67	0	33	0	0	0	0	0	0	
Ngalau Sampit	Reconsum18-71	0	93	0	6	0	1	0	0	0	0	1508
	Reconsum18-54	0	83	0	10	0	7	0	0	0	0	29
	Reconsum18-48	0	84	0	16	0	0	0	0	0	0	128
	Reconsumt18-72	0	83	0	6	0	2	7	0.9	1	0.1	1664
	Reconsum18-51	0	39	0	4	0	14	39	4	0	0	23
	Cave average	0	76.4	0	8.4	0	4.8	9.2	0.98	0.2	0.02	

The shape classifications are adapted from Stoops^45^ and Mackenzie et al.^46^. The average results in each individual cave is calculated below each set of subsamples.

**Table 3:** The mean maximum length and width, and the slope and slope direction of the internal clasts of the thirteen discrete breccia subsamples.

Cave site	Subsample	Mean max. length in mm	Mean max. width in mm	Average orientation	Average slope direction
Lida Ajer	Reconsum18-53	6.21	4.51	12.14N	28.6W
					51E
	Reconsum18-52	1.02	0.83	40.6N	50.3W
					73E
	Reconsum18-50	3.47	2.2	50N	26.57E
					20.94W
	Reconsum18-47	1	0.75	7.4S	25.6E
					27.5W
	Reconsum18-46	4.91	3.21	10S	26.7E
				50N	27.5W
Cave average		3.322	2.3	38.04N	30.96W
				8.7S	40.57E
Ngalau Gupin	Reconsum18-73	14.26	5.82	1.17S	25.9W
	Reconsum18-55	0	0	0	0
	Reconsum18-49	8.7	6.3	17.7 S	45.7W
Cave average		7.65	4.04	9.44S	35.35W
Ngalau Sampit	Reconsum18-71	1.24	1.03	29.1N	38.2E
				47.5S	26.27W
	Reconsum18-54	1.9	1.2	48.4N	50.6W
					51.5E
	Reconsum18-48	1.84	1.2	81.4N	51.4W
				70S	37.2E
	Reconsum18-72	2.57	1.83	2.38N	37.7W
					51.8E
	Reconsum18-51	10.9	7.4	2.2N	21.3W
					24E
Cave average		3.69	2.53	32.7N	40.24E
				58.75S	40.76W

The average result for each individual cave is provided after each set of breccia subsamples. More than one average slope direction is given for subsamples in which clasts commonly slope in two or more cardinal directions.

## Results

Herein, descriptions and interpretations of the samples in this study are combined for fluent communication of the scientific evidence.

### Lida Ajer

The deposits from Lida Ajer are sedimentary remnant deposits cemented to the limestone cave walls and floors. Fractured angular allogenic clasts, predominantly of limestone fragments are cemented into all the matrices. Breccia deposits from LA-1 and LA-4 below the flowstone contain vertebrate remains, solely composed of bone fragments. Breccia LA-3 contains a single vertebrate specimen, an isolated tooth. Host limestone fragments in breccia from LA-2A contain invertebrate fossils, namely crinoids and brachiopods.

### Ngalau Gupin

Like Lida Ajer, deposits from Ngalau Gupin are clay-rich breccia cemented to the limestone cave walls and floors. Unlike Lida Ajer, there are no fractured angular clasts evident in any of the samples. There are no vertebrate remains in sample BC-2A and only a single splinter of bone on the surface of sample NG-A from the cave wall. However, several bone fragments and a single isolated mammal tooth occur in sample NG-A from the cave floor.

### Ngalau Sampit

Samples BC-2 and BC-3 still had significant water retention after drying, obscuring the internal characteristics of the samples. No vertebrate remains occur in samples BC-1, BC-3 and BC-4. A single splinter of bone is evident on the exterior matrix of BC-2. SP-1 includes several isolated bone fragments and is the only sample in Ngalau Gupin, and the entire study, to have a mollusc shell incorporated into the matrix.

Here, we describe in detail the diagnostic characteristics of an individual representative sample from Lida Ajer. For all other sample descriptions please refer to the Supplementary Information.

### Reconsum18-53 LA-1 Lida Ajer

**Figure 7:**
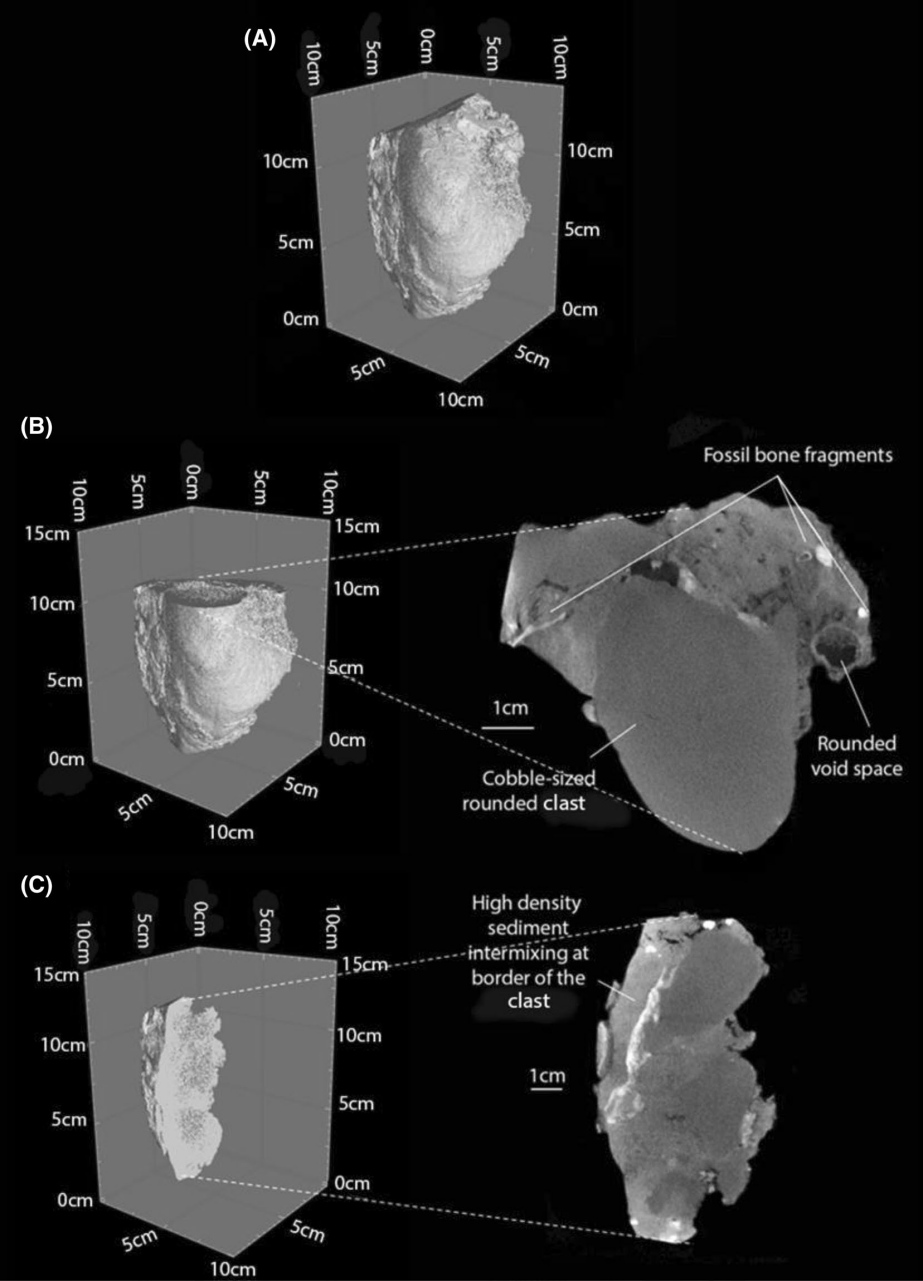
(**A**) Three-dimensional tomographic reconstruction of breccia sample Reconsum18-53; (**B**) Two-dimensional slice section through the radial plane showing the round cavity space; (**C**) Two-dimensional slice section through the tangential plane cut 2.6 cm from the western periphery of the sample at the contact between the cobble and surrounding matrix in (**B**) showing several fossil fragments.

This sample consists of massive sandstone containing a singular cobble and sparse granules consisting of fine-grained limestone (Supplementary Fig. S1). None of the clasts are in contact, and ‘float’ throughout the matrix in an irregular arrangement. Sixty-two percent of the clasts are rounded, and the smaller clasts are very poorly sorted. Hence, this sample is a paraconglomerate. A round cavity, 1.3 mm in diameter is evident in the matrix (Fig. 7B). This cavity is bound by a higher-density border 3 mm in width and is partially filled by lower-density silt. The source of the cavity is difficult to establish; it may be a site where organic matter was once present and has since rotted away. Several fossil bone fragments are interspersed throughout the matrix (Fig. 7C). The fragments are elongate with rounded peripheries and vary from 1 to 12 mm in length and 2 to 8 mm in width. The homogeneous rounding on the peripheries of the incorporated vertebrate remains are consistent with abrasion^37–39^. The overall facies of this deposit is an oligomict extra-formational paraconglomerate. The clasts in sample LA-1 from Lida Ajer lies on a plane 4°–35° north with a bimodal slope direction east between 9° and 46° and west between 9° and 69°, respectively.

## Discussion

### Lida Ajer

The fabrics of LA-1 are interpreted to have formed by the brecciation of host limestone that has been transported short distances within the site of deposition and redeposited with a sandstone matrix by fluid or sediment gravity flow. LA-2A was formed by the collapse of older limestone host rocks from the cave walls and floor due to erosion and re-deposited in a mudstone matrix by fluid or sediment flow. The chaotic fabrics of LA-3 are formed by the rapid flow of poorly sorted thick, muddy slurry of autochthonous and allochthonous material. We suggest the fabrics of LA-4 above the flowstone formed by the alternating wetting and drying of the cave environment during a period of lesser precipitation levels. Similarly, LA-4 below the flowstone formed by the breakdown of host limestone rock that was eroded and redeposited with fine clay-rich sediment. The limestone was saturated by fluid flow and then underwent dissolution.

The classification of Lida Ajer samples is much more diverse and complex than the basic composition and broad typology commonly designated to tropical karst deposits^40^. Two of the deposits, LA-1 and LA-4 above the flowstone were previously misclassified as breccia, but the samples are proven to be paraconglomeratic, suggesting some content of water transported abraded clasts. Samples from LA-1, LA-2A and LA-4 contain clasts derived from the mechanical breakdown of host limestone and other rocks from within the cave. The outlier is sample LA-3, formed by the rapid mass movement of water-laden loose allochthonous debris. When relating the clast orientations to the passages upon excavation, the direction to which the clasts slope within the deposits (including LA-3) are consistently between 3° and 49° north in Lida Ajer cave. The slope of the deposits is consistently east–west though varying considerably in intensity from 9° and 54° and 9° to 68°, respectively. These steep slopes likely reflect the formation of depositional cones in the cave through time, as fluvial deposits are expected to be more horizontal. Most cave breccias occur as conical deposits^41^—so all surfaces would slope in some direction where any bedding could be discerned.

The separation of clasts and matrix indicates periods of shrinkage and swell in all four samples occurs because of differential sediment settlement during wetting and drying and shrinkage of unconsolidated clay-rich soils^42, 43^. Once the rock is cemented with calcite—the degree of shrinkage and settling is reduced. LA-1, LA-2A, LA-3 and LA-4 below the flowstone are indurated rock, hence the wetting and drying process occurred before the samples were cemented. LA-4 above the flowstone is still friable and therefore the process still may be occurring seasonally. The primary agents responsible for clastic deposition in Lida Ajer cave are likely water action and sediment gravity flow. The basic mechanism of deposition for the incorporated vertebrate remains is limited to localised short-distance water transport or sediment gravity flow over a relatively short timescale, although this may not be the case with the rock clasts.

### Ngalau Gupin

The poorly lithified nature of the NG-A floor sample and the primarily soil-grade composition suggests that this deposit may be formed from the disintegration of older consolidated deposits or is a younger deposit that has not yet undergone cementation. The absence of large or complete bones and dominance of isolated teeth in this sample could suggest a significant transport progression of the vertebrate remains in the deposit over a considerable time scale. However, significant transport progression is not the only process that can create this bias of isolated dental remains. Prevalently in Southeast Asia, rodents, and in particular porcupines, are renowned accumulators of bones and teeth. Smith et al.^44^ provide evidence for porcupine gnawing, as well as porcupine fossils, at Ngalau Gupin. In the NG-A wall sample, the high-density sediment appears to have pervasively invaded the structural failures of the matrix in the breccia, forming possible conduits for mineral laden waters that subsequently led to calcite cement precipitation. Unlike Lida Ajer, the composition and texture of NG-A from the cave floor and wall from Ngalau Gupin make these samples breccias. Both breccias have mudstone matrices, but the samples contrast in that NG-A from the cave floor is sourced from the enclosing cave limestone and contains fossil tooth and bone clasts, whereas NG-A from the cave wall is derived from several source rocks outside of the cave site and only a single bone fragment.

As BC-2A consists primarily of speleothem and the central deposit lacks large clasts, it is more difficult to interpret the depositional history of this sample. Thin layers of speleothem, as seen in the neutron scans, are formed by cave drip water. The sudden cease in deposition of speleothem and transition to sandstone in the middle of the sample might indicate a drier period, just as the re-occurrence of speleothem above the sandstone could signify a change to greater precipitation. Interruption of the speleothem may also be due to a large deposit of sediment being washed into the cave. The primary agent in the formation of the breccia deposits in Ngalau Gupin cave is likely water action or sediment gravity flow. The sample lithologies suggest that the basic mechanism of deposition for the incorporated vertebrate remains is limited to localised short-distance water transport or sediment gravity flow over a prolonged timescale.

### Ngalau Sampit

Ngalau Sampit samples have a uniform typology, although the classifications vary as the fabrics of the samples are diverse. Two of the deposits, BC-3 and SP-1 were previously misclassified as breccia, but the samples are paraconglomeratic. The samples from Ngalau Sampit are all muddy sandstones, and all of the samples except SP-1 are intra-formational deposits. The cylindrical features of BC-1 are the sole representation of the plant-life washed into the cave during speleogenesis. The matrix has pulled away from the clasts, which suggests shrinkage due to wetting and drying periods.

The primary agent in the formation of the breccia deposits in Ngalau Sampit cave is likely sediment gravity flow as deposition of incorporated clasts and inclusions was limited to localised short-distance movement and minor water transport. The slopes of the clasts may indicate that the deposits represent part of two or more cones with differing slopes and slope directions.

### Method evaluation

Thermal-neutron scanning has proven an excellent method for revealing the internal composition and structure of the samples, revealing diagnostic internal characteristics that give insights into the taphonomic and formational history of these fossiliferous cave deposits. This is the first time that neutron tomography has been used to conduct a high-throughput survey of geological samples to extract prehistoric data, with a deliberate aim being to achieve minimal residual radioactivation of specimens, necessary for subsequent study. The neutron scans allow visualisation of the spatial distribution of materials where the samples themselves are too poorly indurated to be cut into slabs to study with the naked eye. Furthermore, in the case of better indurated samples, the neutron scans can aid in guiding sawing so as not to cut through a valuable fossil or flowstone deposit. Using NT has allowed significant extraction of important geological and palaeontological information from materials that have high attenuation differences. This approach has allowed elucidation of pre-, syn- and post-depositional histories preserved in the breccias and association with the taphonomic history of the fossil remains incorporated within. Sedimentological analysis of the samples from Lida Ajer, Ngalau Gupin and Ngalau Sampit indicate that significant variation in composition and texture of fossil-bearing cave deposits can occur at the scale of individual cave passages and deposits, resulting in highly complex fossil assemblages that do not allow immediate palaeoecological and faunal interpretation.

### Potential implications

When studying the surface of cave breccias, commonly little to no stratigraphy can be distinguished in the field. This is in part due to the homogeneous appearance of the deposit surfaces and in part as only remnant deposits remain. Furthermore, excavation of the breccia commonly destroys the contextual evidence needed to deduce the depositional history. Neutron tomography has proven an excellent research method to observe the internal geometry of the deposits. In particular, the three-dimensional aspect of NT allows us to visualise and record the slope and slope direction of internal clasts, and thus any internal stratigraphy.

**Figure 8:**
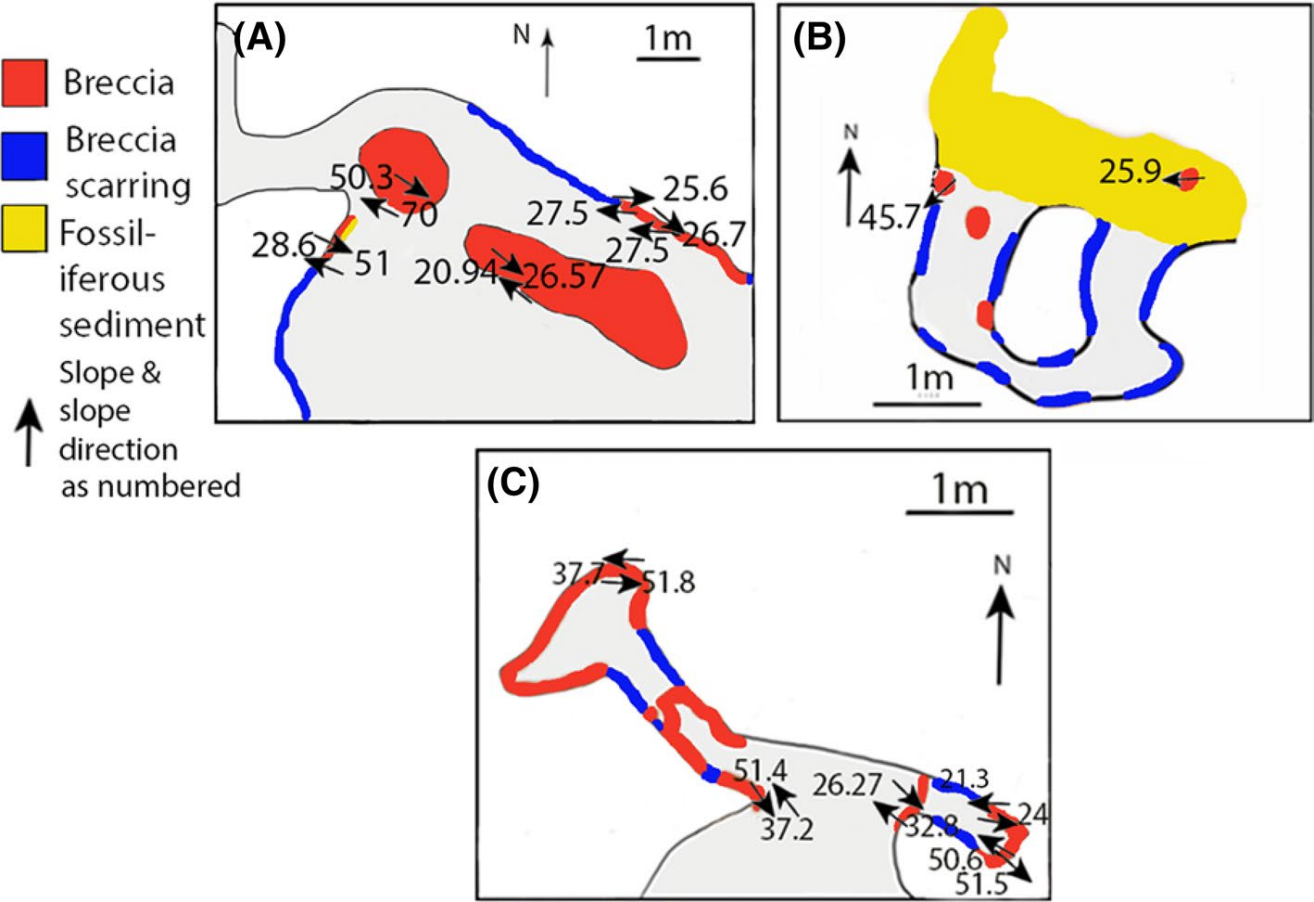
(**A**–**C**) The average slope and slope direction of the internal clasts of the breccia subsamples in relation to the cave passage orientations, mapped directly onto the colour scale cave profile section from (**A**) Lida Ajer cave; (**B**) Ngalau Gupin cave; (**C**) Ngalau Sampit cave. The map symbol shows slope direction as an arrow with the angle in numbers.

In the Lida Ajer samples, the clasts in beds formed in wetter periods predominantly have an easterly slope direction. In beds formed in drier periods, the clasts predominantly have a westerly slope direction. The bimodal nature of a sample may typically reflect cone formation, but these data suggest this is not definitive. Cones form under roof collapses, as debris forms a pile from a central point source. To be a cone, the slope covers the full 360°, it just depends on where the sample is taken and the size of both sample and cone. It can be determined if there was one large cone centred in the fossil-bearing chamber or numerous distinct cone formations as the slope directions in each single rock sample would have formed on a single slope of the cone(s) and therefore have a distinct range of orientations. However, as these bimodal slope directions are from singular rock samples of small size, it is unlikely this reflects cone formation. It is plausible that the opposing slope directions reflect the catastrophic flooding and receding of water during speleogenesis, though the sample size means this cannot be confirmed. Comparing the sedimentological context with these data seems to be important in the overall analysis. Such a configuration appears to reaffirm that these slope directions are dictated by inflow and recession of water, which corresponds with the hypothesis that the deposit formed due to the in-wash of surficial material. It is not possible from this directional data to determine if the vertebrate remains were re-worked from within the fossil-bearing chamber, from a neighbouring chamber or outside the cave. The slopes of LA-2A and LA-3 are strikingly similar, as are the slopes of LA-1 and LA-4 samples (Fig. 8A). These data may act as further indication of two different depositional modes.

Only two of the three samples from Ngalau Gupin, the NG-A wall and floor exposures, contain clasts. The comparative slope and slope direction of two samples with such different compositions suggests there is a shared transport history but there are not enough samples across the cave site, and too little spatial variation between the samples to make any further assessment (Fig. 8B).

When comparing the sedimentological context of rounded, host limestone clasts and the shallow opposing slope directions in Ngalau Sampit breccia samples, the configuration appears to be dictated by inflow and recession of water, which corresponds with the hypothesis that the limestone clasts are eroded and re-worked by water action. It is not possible from this directional data to determine if the vertebrate remains were re-worked from within the fossil-bearing chamber, from a neighbouring chamber or outside the cave. The slope and slope direction of clasts in BC-1 must be considered with caution as the internal analysis suggests the sample has evidently been heavily re-worked by flooding into the cave during speleogenesis. Furthermore, only the peripheries of BC-2 and BC-3 sample are visible due to neutron-attenuation issues, and so the slope directions retained from the margins of the deposits must be considered with the same caution. SP-1 sample is most likely a horizontal deposit formed from the eroded remnants of a cone (Fig. 8C).

Neutron tomography data give an approximate hypothesis as to the transport and depositional history of the breccia samples. All data—slopes, rounding or angles, compositions, etc., is needed to reconstruct the stratigraphy of breccia deposits. Should the method be developed further, the depositional contextual evidence stored within the breccia is crucial. The key is to recognise any stratigraphy within that may otherwise look homogeneous, and neutron scan data helps in this effort as the data derived from this method are three-dimensional. The results of the orientation data are considered tentatively as relative to the original whole deposit, and even the remnant breccias that remain, the samples may be too small to form a full reconstruction of such a complex process. It may help with this concern to use more, and larger, samples using the same facilities with either higher neutron energies or the same neutron energies and much more intensive drying methods to reduce neutron absorption issues. Moreover, the number of clasts in each sample varies greatly from none into the thousands. Additionally, should the samples reflect cone formation, a whole cone has 360° of clast slope variation to study, but in one small sample this only reflects certain slope directions. However, these are interesting results that show there is potential to reconstruct the stratigraphy of tropical breccia deposits in future studies. We recommend the excavation of additional samples across the cave site, multiple samples from each discrete breccia site, and a larger sample size for a comprehensive stratigraphic reconstruction.

## Conclusion

This study has successfully recorded key internal diagnostic characteristics of thirteen cave ‘breccia’ samples from Sumatra, Indonesia using high-throughput thermal-neutron tomography as a proof-of-principle means of surveying a large collection of geological samples. This analysis was undertaken without destructive sample preparation or extraction and resulted in only very brief radioactivation of samples, enabling their clearance and return within 1 week of measurement. Our novel methodology suggests there is a great deal of evidence stored in the complex internal structures of these breccias at the macroscopic and microscopic levels. The spatial information derived from the three-dimensionally rendered scans provides a high resolution taphonomic and depositional analysis of the vertebrate-bearing breccia deposits. These three-dimensional data from Ngalau Gupin, Lida Ajer and Ngalau Sampit characterise the diagnostic signature of the deposits to determine breccia type. Defining breccia type provides critical information about the depositional conditions and history of the deposit. Thus, classifying a breccia has acted as direct indication of the basic mechanisms and timescale of accumulation of incorporated vertebrate remains, and the interactions with the depositional environment. When applied to the wider scope of research, this study has the potential to resolve stratigraphic provenance and temporal positions of fossil-bearing deposits in complex depositional environments in Southeast Asia and around the world. Not only could analysing spatial distribution data help constrain stratigraphy and confirm fossil contemporaneity, the biostratigraphical framework that can be created provides higher resolution analysis of palaeoenvironment and the agents responsible for site formation events. The deposits may be too friable to analyse any other way, and so neutron tomographic imaging is critical to this research.

## Supplementary Information

Supplementary Information 1.

Supplementary Information 2.

## Data Availability

All data generated or analysed during this study are included in this published article (and its Supplementary Information files).
