# Supplementary material for: High-resolution high-throughput thermal neutron tomographic imaging of fossiliferous cave breccias from Sumatra
Source: Sci Rep. 2021 Oct 7;11:19953. doi: 10.1038/s41598-021-99290-0 (PMC8497489; doi:10.1038/s41598-021-99290-0)
Supplement: Supplementary file 1 — Supplementary Information 1. [file 41598_2021_99290_MOESM1_ESM.docx]

**High-resolution high-throughput thermal neutron tomographic imaging of fossiliferous cave breccias from Sumatra**

Holly E. Smith, Joseph J. Bevitt, Jahdi Zaim, Yan Rizal, Aswan, Mika Rizki Puspaningrum, Agus Trihascaryo, Gilbert J. Price, Gregory. E Webb, Julien Louys

Holly E. Smith **Corresponding author e-mail* @ *holly.smith11@griffithuni.edu.au* *-* *Australian Research Centre for Human Evolution, Environmental Futures Research Institute, Griffith University, Brisbane QLD 4111, Australia*

Joseph Bevitt @ *joseph.bevitt@ansto.gov.au - Australian Centre for Neutron Scattering, Australian Nuclear Science and Technology Organisation, New Illawarra Rd, Lucas Heights NSW 2234, Australia*

Jahdi Zaim @ *zaim@gl.itb.ac.id* - *Geology Study Program, Institut Teknologi Bandung, Jawa Barat 40132, Indonesia*

Yan Rizal @ *yan@gl.itb.ac.id* - *Geology Study Program, Institut Teknologi Bandung, Jawa Barat 40132, Indonesia*

Aswan @ *aswan_gl@gl.itb.ac.id* *- Geology Study Program, Institut Teknologi Bandung, Jawa Barat 40132, Indonesia*

Mika Rizki Puspaningrum @ *mika-rizki@gl.itb.ac.id* - *Geology Study Program, Institut Teknologi Bandung, Jawa Barat 40132, Indonesia*

Agus Trihascaryo @ *agusgeo@yahoo.com* *Geology Study Program, Institut Teknologi Bandung, Jawa Barat 40132, Indonesia*

Gilbert J. Price @ *g.price1@uq.edu.au* - *School of Earth and Environmental Sciences, The University of Queensland, Brisbane QLD 4072, Australia*

Gregory. E Webb *@ g.webb@uq.edu.au - School of Earth and Environmental Sciences, The University of Queensland, Brisbane QLD 4072, Australia*

**Supplementary Information**

*Reconsum18-52 BC-1 LA-2A Lida Ajer*

This sample consists of two angular cobble-size blue-grey fine limestone clasts within a calcareous mudstone. The limestone clasts have an imbricated fabric, but the specimen is too small to determine if that reflects the overall breccia. It is unlikely that this imbrication is caused by flowing water, it is probable this uniform orientation reflects deposition on a slope as the overall facies reflects a collapse deposit and the sample is not comprised of uniform beds. More data are required to confirm the catalyst of this imbrication as the samples are too small for a definitive interpretation. The largest clast contains brachiopod and crinoid moulds (Supplementary Figure 2) that are consistent with the host limestone of Permian-Carboniferous age (Gupta, 2005 [40]; Louys et al. 2017 [14]). A fragment of speleothem also occurs. Large cavities occur between the larger clasts and speleothem – consistent with a collapse breccia. The speleothem fragment has diagnostic growth layers that are visible as variations in neutron apparent density in cross section (Figure S14C). The small dense pebble-size discoid clasts in the matrix appear to be of similar size, but the sample itself is not well sorted (Figure S14B). The discoid clasts comprise approximately ten percent of the matrix. A single tooth of 1.3 cm in length and 0.4 cm in width with two elongate roots is embedded in the matrix (Figure S14D). The internal fabrics observed in LA-2A indicate that the overall facies of this deposit is a monomict intra-formational collapse breccia. In relation to passageway orientation in the field, the internal clasts are orientated 20-49° north, sloping west between 38-63°, except for the speleothem fragment which slopes 73° east.

*Reconsum18-50 BC-1 LA-3 Lida Ajer*

This sample is a massive, very poorly sorted indurated sandstone with no apparent bedding or structure, containing large angular limestone clasts (Supplementary Figure 3). There is one cobble-sized silt-grade angular orange clast of stained limestone. All remaining clasts are fine-grained angular blue-grey pebble-sized limestone. Tomographic analysis reveals a considerable array of clasts from angular cobbles to coarse sand grains (Figure S15B). Clasts vary in shape including tabular, equant, irregular, rounded and discoidal. Individual clasts range from dark grey to white in the neutron scans, reflecting variable neutron apparent density. Several cavities ranging from 1 to 10 mm in size occur in the matrix. These cavities are round or elliptical in shape and contain small, ~round, high-density clasts between 1 and 4 mm in diameter (Figure S15C). No fossils are evident in this sample. Higher and lower density sediment is mixed throughout the matrix. The large variety in composition and high proportionality of incorporated angular clasts observed in LA-3 suggests that this sample best represents a chaotic oligomict breccia that formed as a debris flow within the passage. In relation to passageway orientation at the point of excavation, the internal clasts of LA-3 are orientated 8-87° north with a bimodal slope direction of 2-52° west and 2-71° east.

*Reconsum18-47 LA-4 above flowstone – Lida Ajer*

This sample is a poorly indurated siltstone comprised of five interbedded fabrics and an abundance of rounded clasts. These features suggest this sample is a water-borne paraconglomerate deposited above a flowstone (Supplementary Figure 4). Fabrics A, C & E are matrix-supported deposits consisting of a fine sand. There are 1 mm cavities randomly interspersed throughout these matrices. Fabrics B & D consist of clast-rich beds with very fine sand matrix and relatively well sorted high-density sub-rounded to angular clasts (Figure S16B). These clasts range in size from <1 - 9 mm and are partly surrounded by a 1 mm thick void. The long axes of these clasts are horizontally aligned. The most distinctive features of Fabrics C and E are distinct bands of low-density fine sediment. Two visible laminations within the fine beds are defined by poorly developed coarse laminae with internally mottled, possibly peloidal texture (Figure S16C). The laminations are 3 mm in height and have 2 mm cavities randomly interspersed throughout. Two larger clasts occur in Fabrics C & E that are 8 cm in size, angular and lower in density. Two rounded fossil bone fragments, 3 mm in size occur in the matrix of Fabric E. Thus, LA-4 above the flowstone is a poorly lithified variably bedded monomict intra-formational paraconglomerate. In relation to passageway orientation at the point of excavation, clasts of LA-4 above the flowstone are orientated 4-19° south with a bimodal slope direction of 2-49° east and 2-54° west, respectively.

*Reconsum18-46 LA-4 below the flowstone – Lida Ajer*

This sample is a heavily lithified mudstone with superficial orange and black staining (Supplementary Figure 5) (Figure S17). Underlying Fabric A is an indurated sandstone, whereas fabric B is an indurated silty mudstone. Surficially, the sample appears to be a breccia due to the presence of a single black fine-grained angular limestone cobble protruding from the surface, but the neutron scan suggests a conglomerate as the internal clasts are predominantly rounded (Figure S17B). Fabric A and Fabric B are separated by a fracture 1 mm in width (Figure S17C). A single low density angular pebble is incorporated into the highly fractured and cemented matrix of Fabric A. White calcite veins 1 mm in width run throughout the clast. Two cavities in Fabric B are lined with mineral precipitates (Figure S17B) and two fossil fragments occur: a single round bone fragment, situated at the base of Fabric B and an elongate fragment that protrudes from the periphery of the deposit into the crack between the two fabrics. The overall facies of LA-4 below the flowstone deposit is a well-lithified oligomict extra-formational conglomerate. The internal clasts of LA-4 below the flowstone have a uniform orientation, consistent with alignment by transport. In relation to passageway orientation at the point of excavation, most clasts are orientated 9-12° south with a unimodal slope direction of 13-49° east. However, the two cobble-sized lower density clasts are orientated 22 and 67° north with slope directions of 17° and 38° west.

*Reconsum18-73 – Ngalau Gupin site NG-A cave floor*

This sample (Supplementary Figure 6) is un-indurated and became considerably more friable after undergoing drying preparation. The sample ranges from massive to more obviously bedded with an irregular texture (Supplementary Figure 6) (Figure S18B). The massive facies with irregular texture are clay-rich muds that vary in neutron apparent density and abundance of cavities. Fabric B has the highest apparent neutron density, followed by fabrics A and C and finally fabric D. Fabric D is heavily fractured by with open cracks and cavities throughout the sediment. There are no larger clasts in this sample other than the abundant vertebrate fossils, including two fossil teeth. The first tooth (Fabric B) is 1 cm in length, round in occlusal view with numerous enamel projections creating an irregular occlusal surface. Numerous high-density bone fragments ‘float’ in the sediment of the more massive facies (Figure S18C). These fragments are elongate and rounded, averaging 10 – 30 mm in size. The second isolated tooth (massive Fabric D) is 20 mm in length and 7 mm in width (Figure S18D). An axial section of the heavily fractured dense deposit reveals that the tooth has two distinct lophs, each segregated by a simple U-shaped infundibulum. The internal fabrics in NG-A floor deposits indicate brecciated clay-rich sediments. In relation to passageway orientation at the point of excavation the clasts are orientated 1-2° south with a unimodal slope direction of 3-60° west. The only exception is the large, isolated mammal tooth orientated 22° south-east and sloping north-west by 2°.

*Reconsum18-55 – Ngalau Gupin Breccia 2*

This sample contains two speleothems interstratified with a fine-grained sandstone (Supplementary Figure 7) (Figure S19). The overlying fabrics (Fabric A & C) are speleothem that laminates or adheres to the surfaces of the central fabric (Fabric B) (Figure S19B). Fabric C is the lowest in density of the three deposits, with a continuous small-scale sequence of horizontal parallel laminations throughout that reflects the sheet-like deposition of the flowstone (Figure S19C). Fabric B is a much higher density massive fine-grained sandstone with no large clasts and no incorporated fossils. The internal fabrics observed in BC-2 indicate flowstone adhering to extra-formational sandstone.

*Reconsum18-49 – Ngalau Gupin site NG-A cave wall*

This sample is comprised of two poorly lithified massive mudstones (Supplementary Figure 8). Despite drying the sample, there was high water retention that suggests a clay-rich composition. There is a notable gradation in colour across the sample from an earthy brown to dark beige and finally a dull lustre white. The first layer (Fabric A) is a mudstone with no evident bedding or structure (Figure S20B). Only three clasts occur in the entire sample, all within Fabric A: an irregular lower density clast 14 mm in length and 8 mm in width, a rounded higher density clast 8 mm in length and width and an angular fossil bone fragment. The angular bone splinter is 9 mm in length and protrudes from the surface of the sample (Figure S20C). Fabric B is a massive fine sandstone that is heavily fractured, disjointed, with numerous higher density clasts clearly separated from the crumbling section on the lower right (Figure S20D). The internal fabrics in NG-A wall deposits suggest an oligomict extra-formational breccia.

*Reconsum18-71 Chamber 1 BC- 1 Ngalau Sampit*

This sample is a friable massive sandstone with two distinct fabrics (Supplementary Figure 9) (Figure S21). Underlying Fabric A is a muddy sandstone with sparse very poorly sorted, sub-rounded clasts randomly interspersed throughout. These clasts make up less than twenty percent of the overall matrix. Fabric A gradually grades into Fabric B, a silty mudstone (Figure S21B). Fabric B has more abundant clasts, approaching being clast supported with numerous poorly sorted, sub-angular high-density clasts randomly interspersed throughout. These clasts make up approximately forty percent of Fabric B. Across the whole sample, abundant cylindrical features cut through the sediment (Figure S21C). The features range from straight to branching ‘Y’ & ‘T’-shaped and curved ‘J’-shaped structures. They vary considerably from 0.5 mm to 2 mm in diameter and 3 mm to 30 mm in length. Approximately one-half of these features consist of void space, while the other one-half is filled by silt. Many of these features intersect one another apparently randomly. These cylinders are bordered by discrete high density angular fragments 1 -2 mm in size representing calcite that was deposited along the inside of each cylinder and then was broken up. The matrix-supported lithology with carbonate clasts preserved in sample BC-1 best represents an oligomict intra-formational breccia. The clasts lie on highly variable bimodal orientations of 3-68° north and 12-82° south. The clasts have a highly variable bimodal slope; east by 3-89° and west by 4-84°. There is no uniform direction or orientation evident in the clasts incorporated into this sample, perhaps related to bioturbation or sudden deposition of material into the cave.

*Reconsum18-54 Chamber 1 BC-2 Ngalau Sampit*

This sample is a muddy sandstone (Supplementary Figure 10). The neutron scan produces mainly null results, as the high attenuation of water content has limited digital content extraction (Figure S22). The retention of water after intensive drying suggests this sample has very high porosity and low permeability. There is more noise on one side of the sample than the other. The sample has a high attenuation due to the thickness and residual water, the thicker the sample the more water is stored within. Also, as aforementioned in the methodology, all samples were imaged with rapid 180° scans rather than 360°, which may also produce the same result. A single rounded fossil fragment occurs on the periphery of the sample within a clast-supported fabric (Figure S22B). The clasts are predominantly rounded and range from 0.3 – 1 mm in diameter (Figure S22C). In relation to passageway orientation in the field, the clasts on the peripheries of BC-2 are orientated 41-79° north with a bimodal slope direction between 49-51° west and 14-89° east.

*Reconsum18-48 BC-3 Ngalau Sampit*

The sample is a muddy sandstone, similar in composition to Reconsum18-54 (Supplementary Figure 11). No evident structure or clasts occur on the block sample exterior. The neutron scan has a similar issue to those of Reconsum18-54 as high attenuation of water content limits digital content extraction (Figure S23). The scan quality is, however, better than that of the previous sample and a single course matrix-supported massive fabric is distinguishable (Figure S23B). This sample is a paraconglomerate comprised of approximately ten percent rounded clasts from 1 mm to 3 cm in size that ‘float’ within the matrix (Figure S23C). The clasts vary in density, including those higher to, similar to, and lower in density than the surrounding matrix. All clasts are similarly surrounded by a 1 to 2 mm void space that separates the clast from the matrix. Several of the larger clasts are fractured into smaller fragments, and the fragments are separated by much larger 4 – 6 mm void spaces. The clasts and inclusions in the periphery of BC-3 are orientated 82-89° north with a bimodal slope direction between 4-84° west and 3-39° east.

*Reconsum18-72 BC-4 Chamber 2 Ngalau Sampit*

This deposit consists of three fabrics: a silty sandstone, a muddy sandstone, and a flowstone (Supplementary Figure 12) (Figure S24). The overlying material (Fabric A) is a clast-supported coarse-grained sandstone with very poorly sorted clasts. The clasts are rounded, relatively spherical and range in size from 1 to 3 mm. No bedding structure is visible in this fabric. The matrix is fine to medium sand visible to the naked eye. There is a sharp contact to a low-density speleothem (Fabric B). This flowstone is massive with no clasts or bedding structures evident (Figure S24B). This material has a sharp conformable contact to the final underlying muddy sandstone fabric (Fabric C). Fabric C is a clast-supported muddy sandstone with clusters of angular clasts from 5 to 30 mm in width. Some of these clasts are clearly fractured in-situ, once amalgamated as singular larger clasts. This could reflect wetting and drying with shrinking clays. This could mean that none of the clasts have been transported, instead formed in-situ by soil-like processes. The material grades from a layer that has no evident fracturing to a layer with moderate fracturing and disintegration of the angular clast within, and finally to a layer exhibiting heavy fracturing and distortion (Figure S24C). The previous morphology of each entire clast can still be indistinctly determined. The original clasts were rounded in shape and were approximately 30 mm in size. This relationship suggests the brittle carbonate clasts underwent localised fracturing in the ductile matrix due to collection and dry-wet behaviour. There is minor shear of the fractured clasts that displaces the fragments, also likely due to wet-dry behaviour. There is minor disjunction of the clast fragments, which are separated by irregularly spaced voids. The voids isolate each fractured fragment from its neighbour and the surrounding matrix. The void spaces vary from 1 to 3 mm in width. Two rounded fossil bone fragments that are 20 mm in diameter are evident. The overall facies in BC-4 is an intra-formational breccia. Fabric C grades upward from a clast-supported to a matrix-supported fabric in which the frequency of clasts is inversely related to their average size. In relation to passageway orientation in the field, the clasts are orientated 1-5° north with a bimodal slope; east by 2-88° and west by 12-87°.

*Reconsum18-51 Chamber 1 SP-1 Ngalau Sampit*

The sample is a paraconglomerate; a fine silty matrix-supported sandstone with both angular and rounded clasts (Supplementary Figure 13) (Figure S25). The clasts are white, orange, and blue grey in colour suggestive of variable origins. The clasts vary from sand s (<1 mm) to granules (>2 mm). The clasts within the matrix are slightly denser than the surrounding material and are complete with no visible fractures or deformation. There are irregular cavities isolating all the angular clasts from the surrounding matrix. The cavities are variably sized from 1 mm to 1 cm in width. The angular clasts have a thin tapering layer of cement that is approximately one millimetre thick on the surface areas of the external clast profiles (Figure S25B). The sample contains two bone fragments and a single gastropod (Figure S25C). The well-rounded bone fragments have a similar width of approximately 10 mm, although the lengths of the fragments are 10 and 30 mm. The gastropod shell is visible under the cement with the outer lip protruding from the sample surface (Figure S25D). As well as the clasts and fossils, there are soil glaebules incorporated into the matrix (Figure S25C). This material contains well-rounded clasts of variable size from >1 mm to 2 mm. The soil glaebules average 60 mm in diameter. There are irregularly spaced cavities averaging 10 mm in width partially isolating these soil glaebules from the surrounding matrix. The overall facies of Sample SP-1 is a polymict extra-formational paraconglomerate. In relation to passageway orientation in the field, the clasts and inclusions are orientated 1-3° north, with a bimodal slope direction between 9-40° and 9-50° west.

**Supplementary Figures**


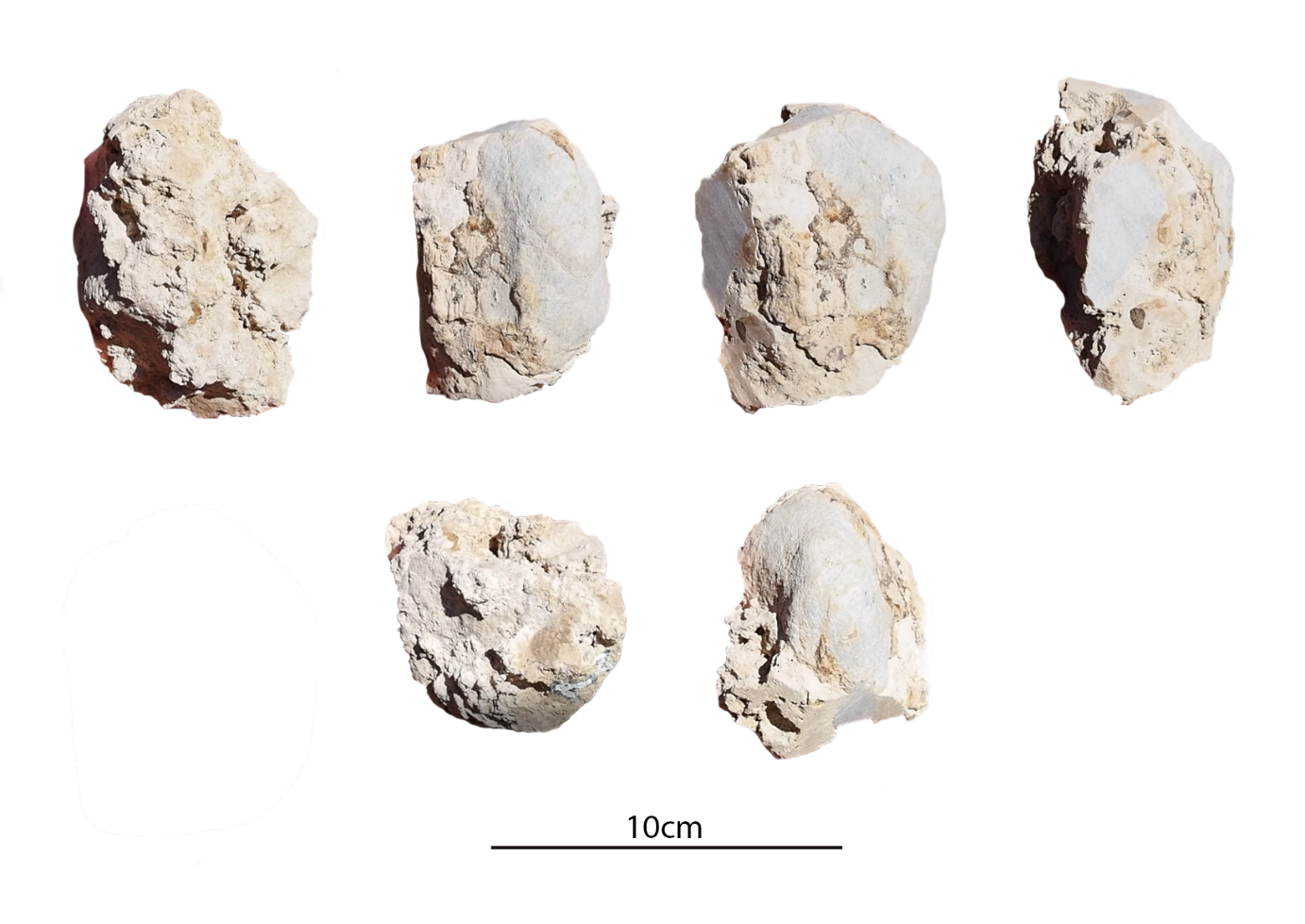


Figure S1. Reconsum18-53 block sample (top left) back facing radial place (top second left) forward facing radial plane (top second right) forward facing transverse plane (top right) downward facing transverse plane (bottom left) north facing tangential plane (bottom right) south facing tangential plane


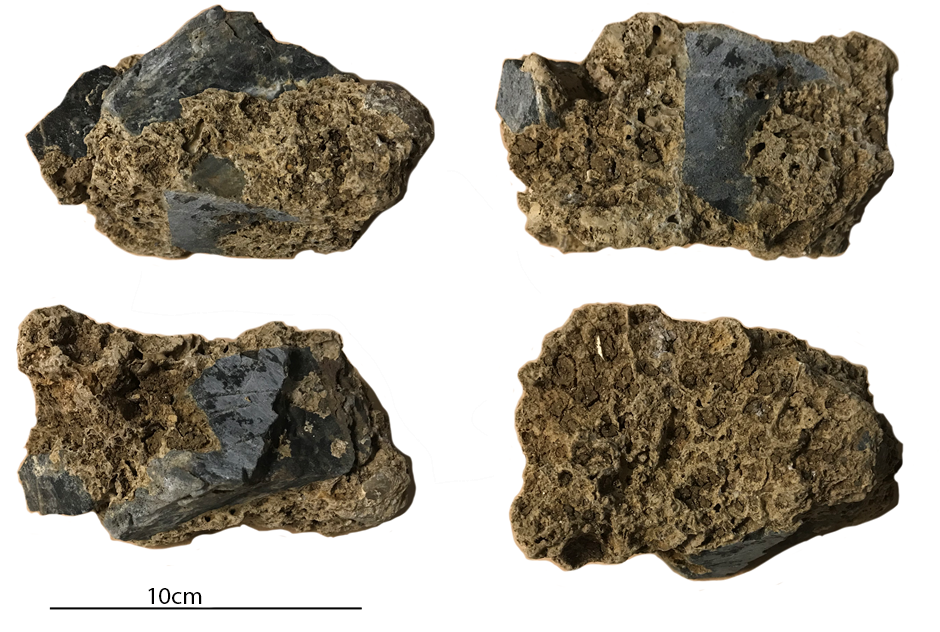


Figure S2. Reconsum18-52 block sample (top left) backward facing radial plane (top right) downward facing transverse plane (bottom left) upward facing transverse plane (bottom right) forward facing north facing radial plane


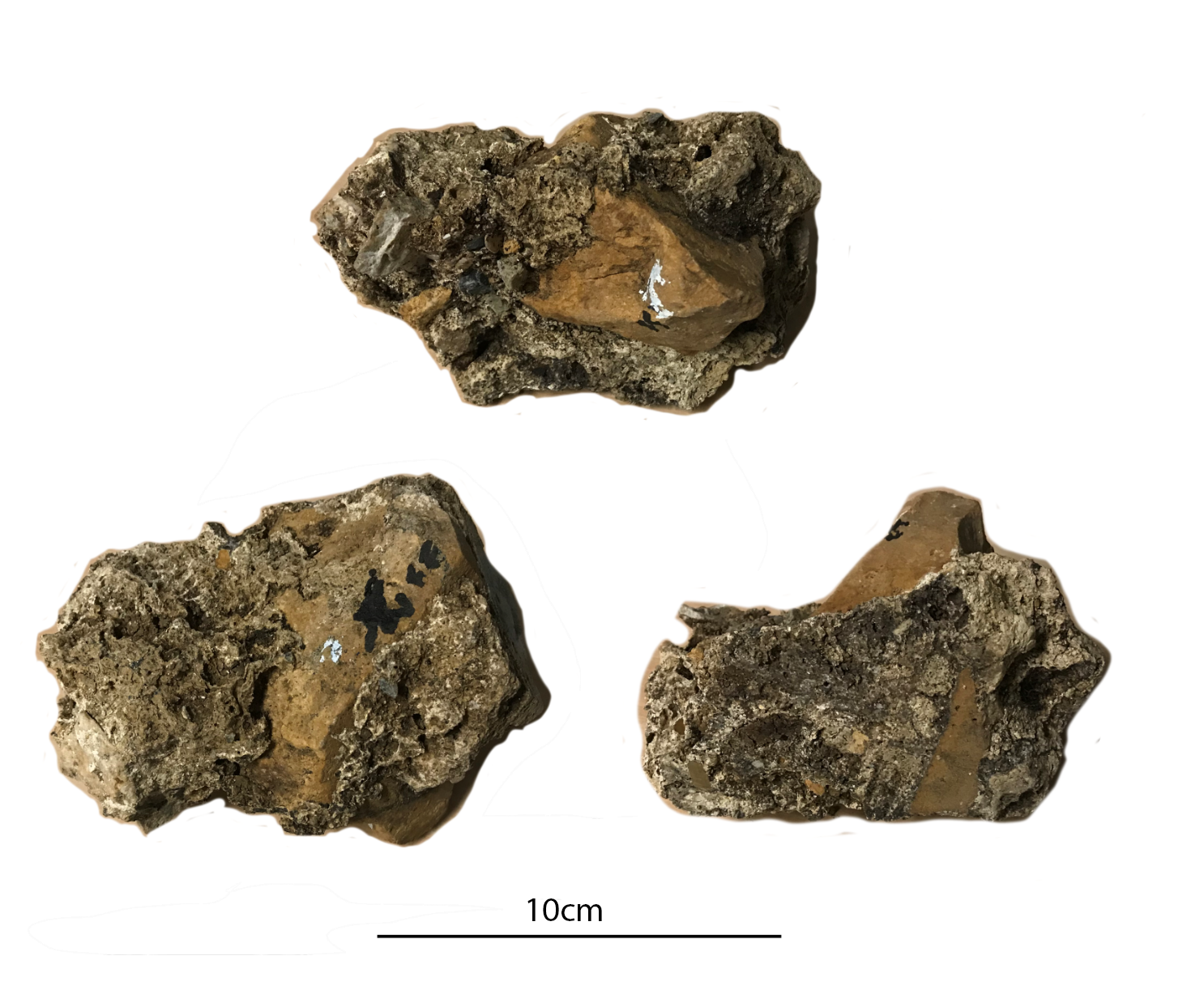


Figure S3. Reconsum18-50 block sample (top) upward facing transverse plane (top right) backward facing radial plane (bottom left) forward facing radial plane (bottom right)


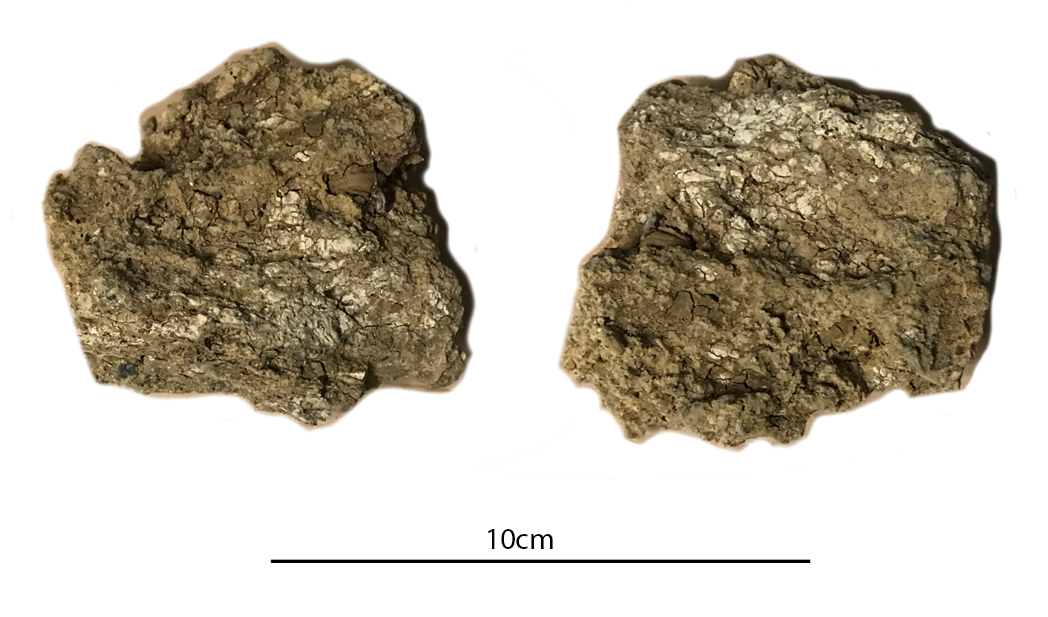


Figure S4. Reconsum18-47 block sample (left) forward facing radial plane (right) backward facing radial plane


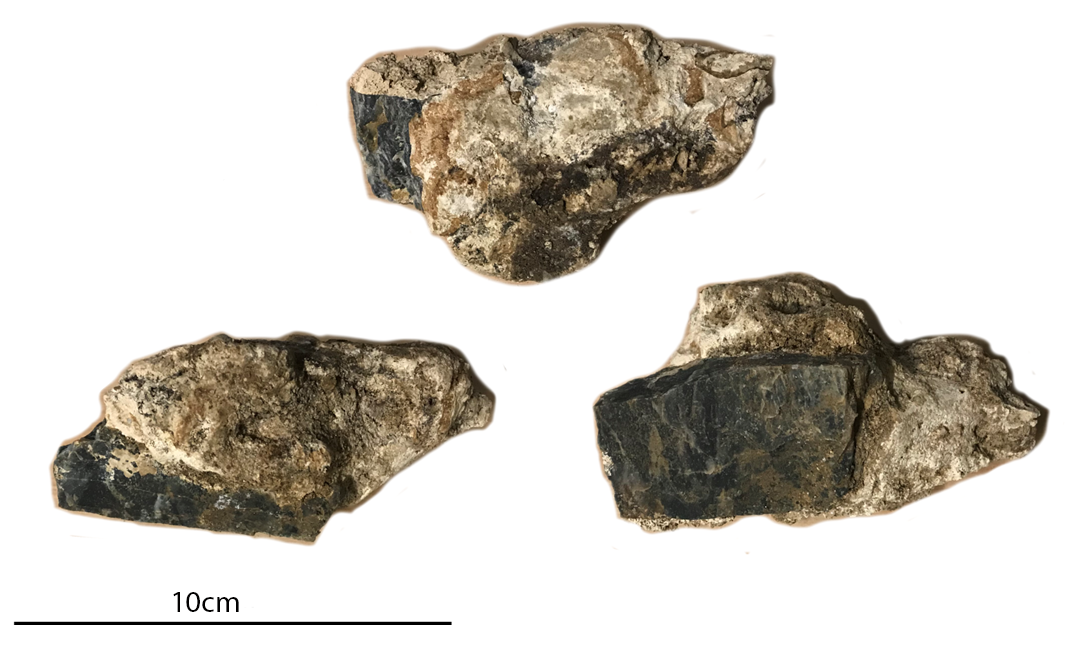


Figure S5. Reconsum18-46 block sample (top) upward facing transverse plane (bottom left) backward facing radial plane (bottom right) downward facing transverse plane


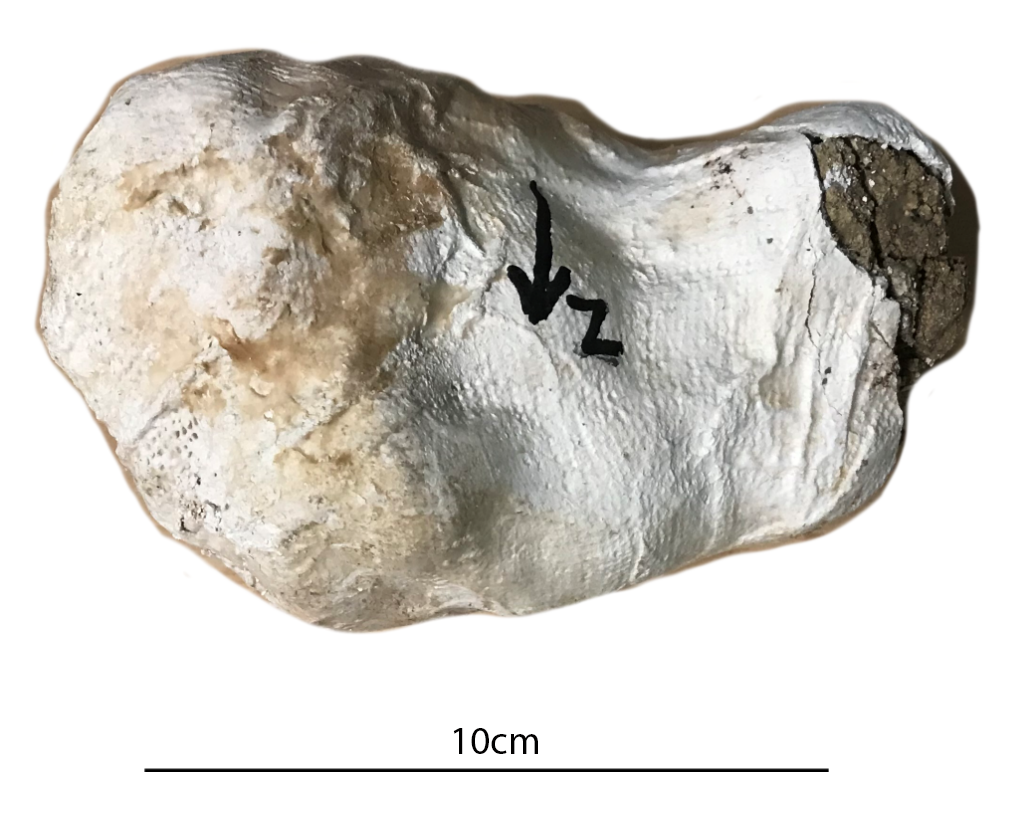


Figure S6. Reconsum18-73 block sample from site NG-A in transverse view, minimal area exposed due to evident friability


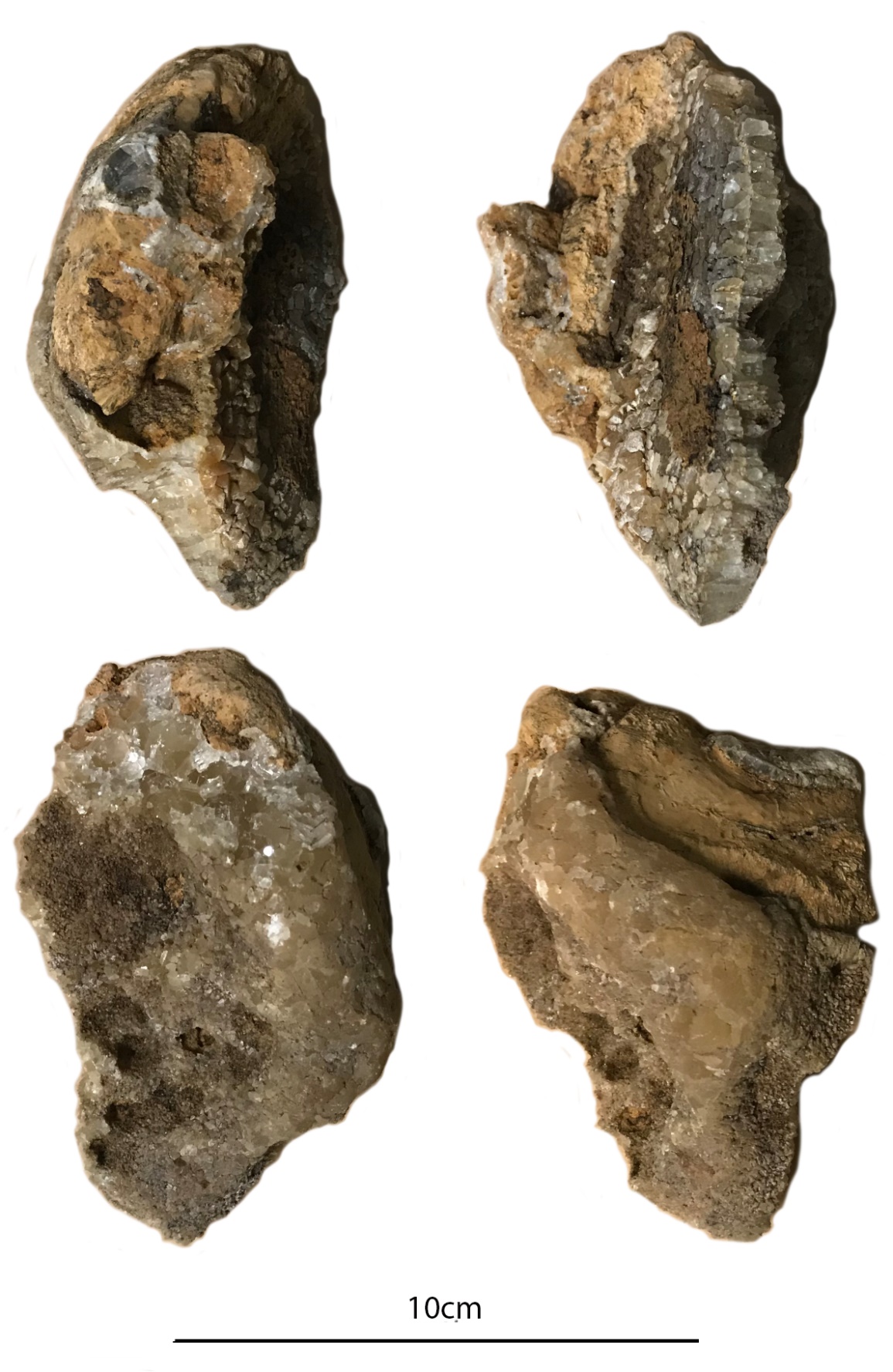


Figure S7. Reconsum18-55 block sample (top left) south facing tangential plane (top right) north facing tangential plane (bottom left) internal radial plane (bottom right) forward facing radial plane


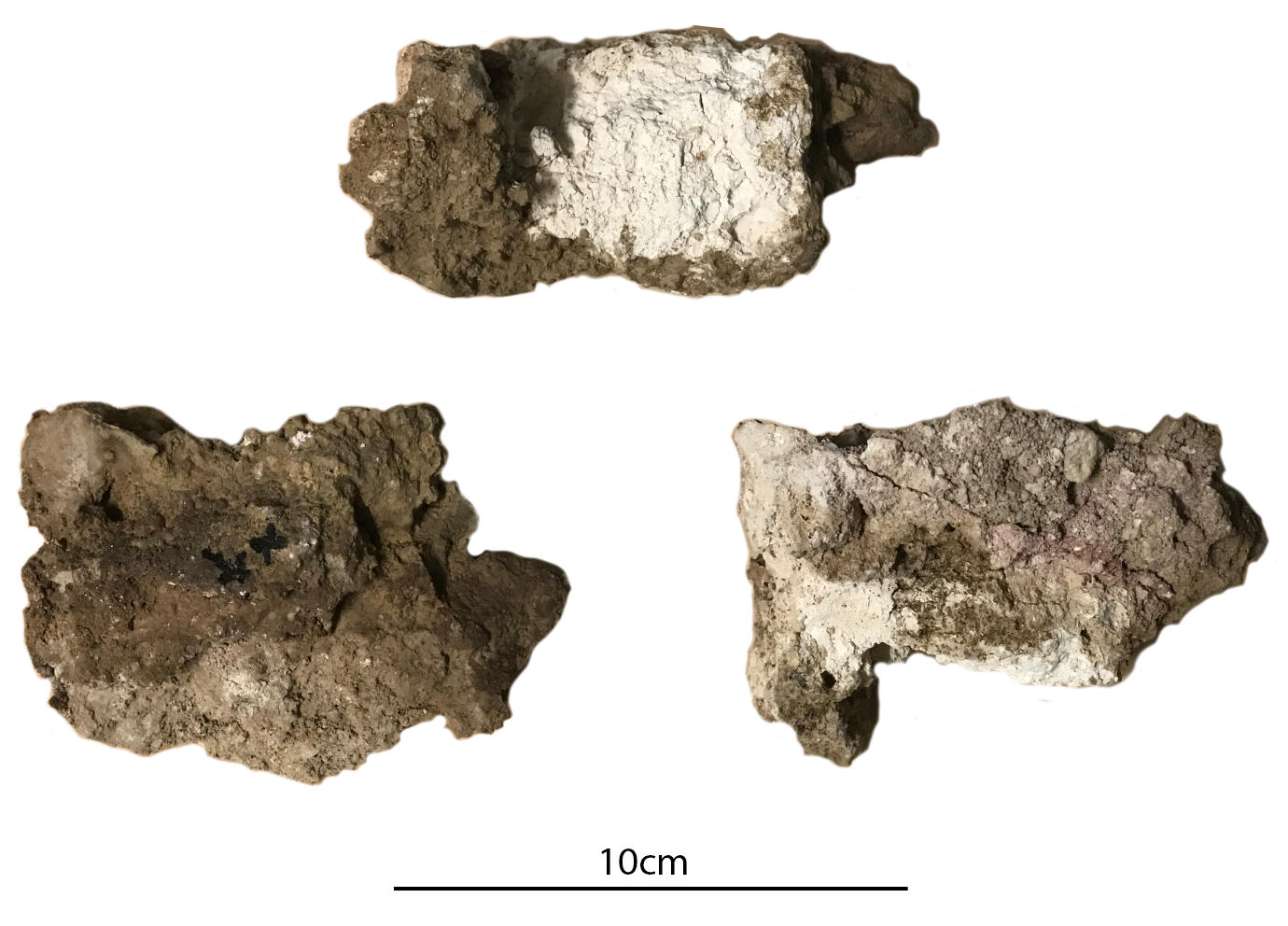


Figure S8. Reconsum18-49 block sample (top) first view tangential plane (bottom left) upward facing transverse plane (bottom right) downward facing transverse plane


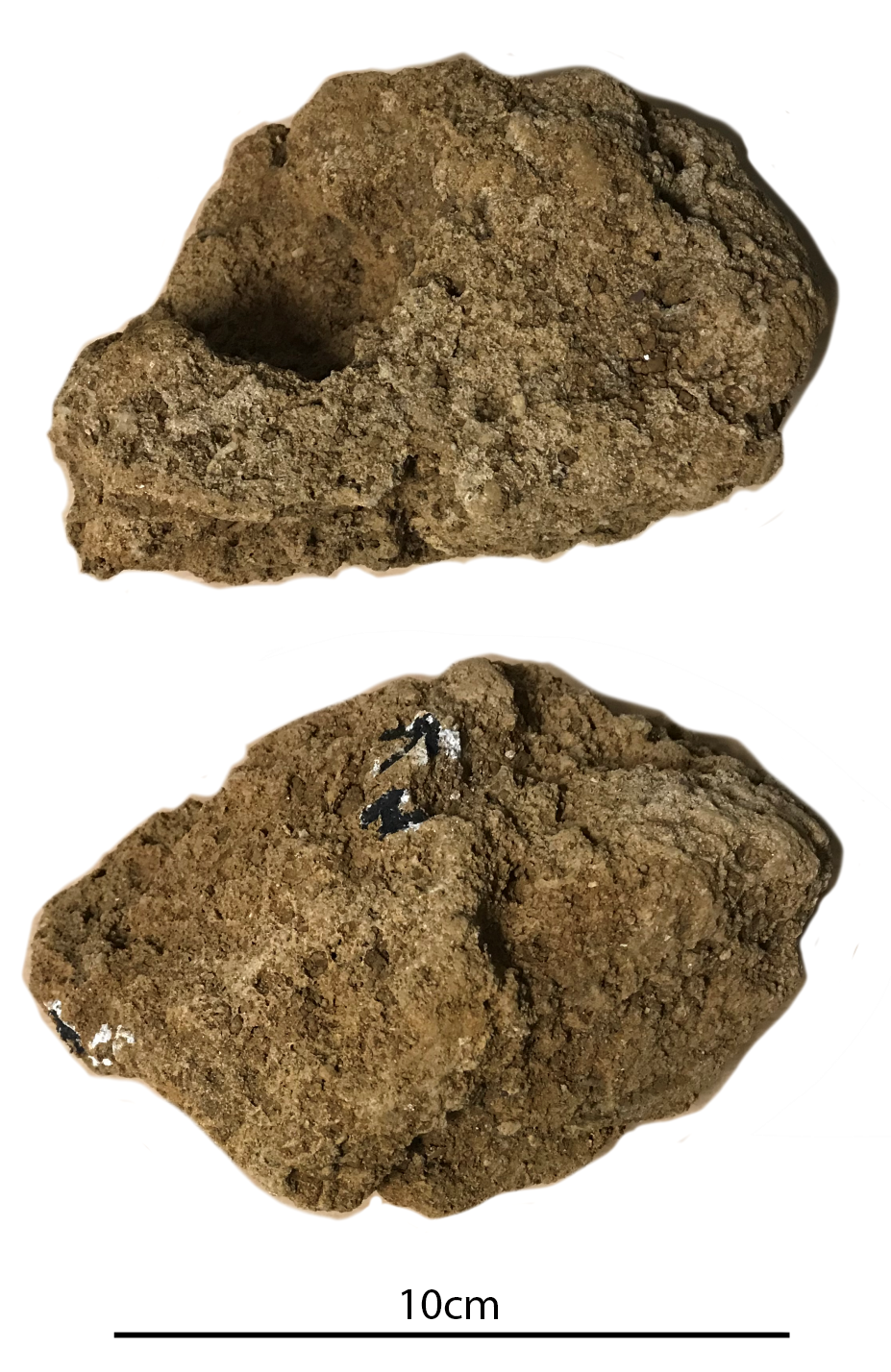


Figure S9. Reconsum18-71 block sample (top) upward facing transverse plane (bottom) downward facing transverse plane


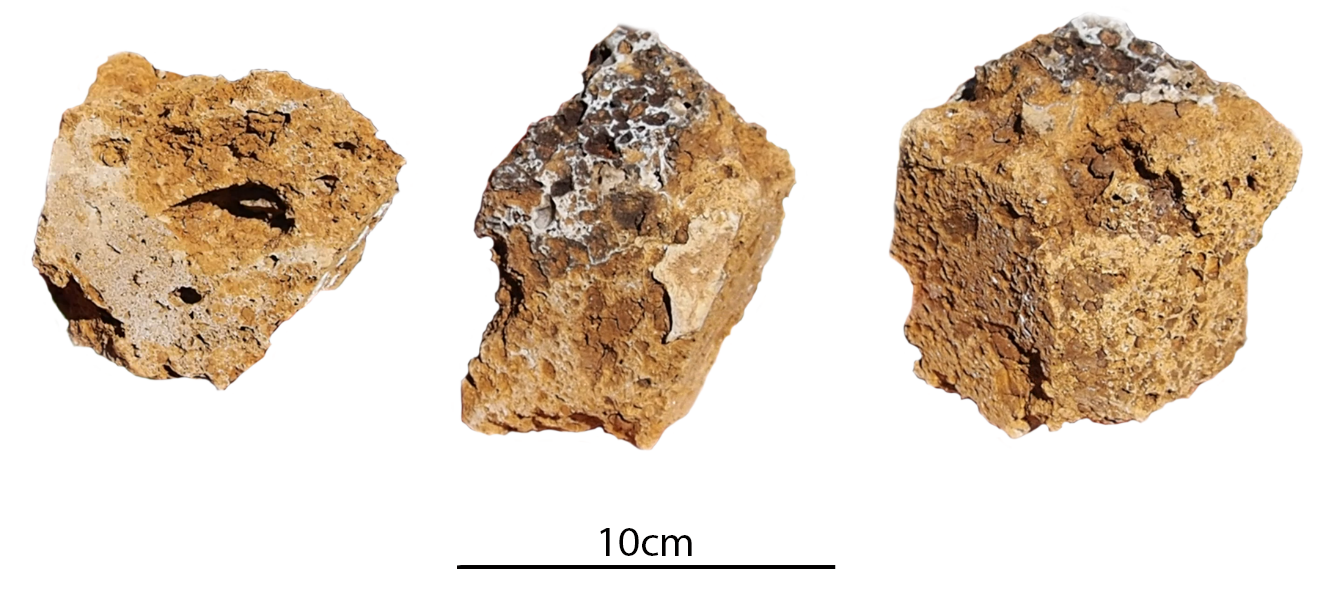


Figure S10. Reconsum18-54 block sample (left) downward facing tangential plane (middle) upward facing tangential plane and backward facing radial plane (right) upward facing tangential plane, forward facing radial plane and south-facing transverse plane


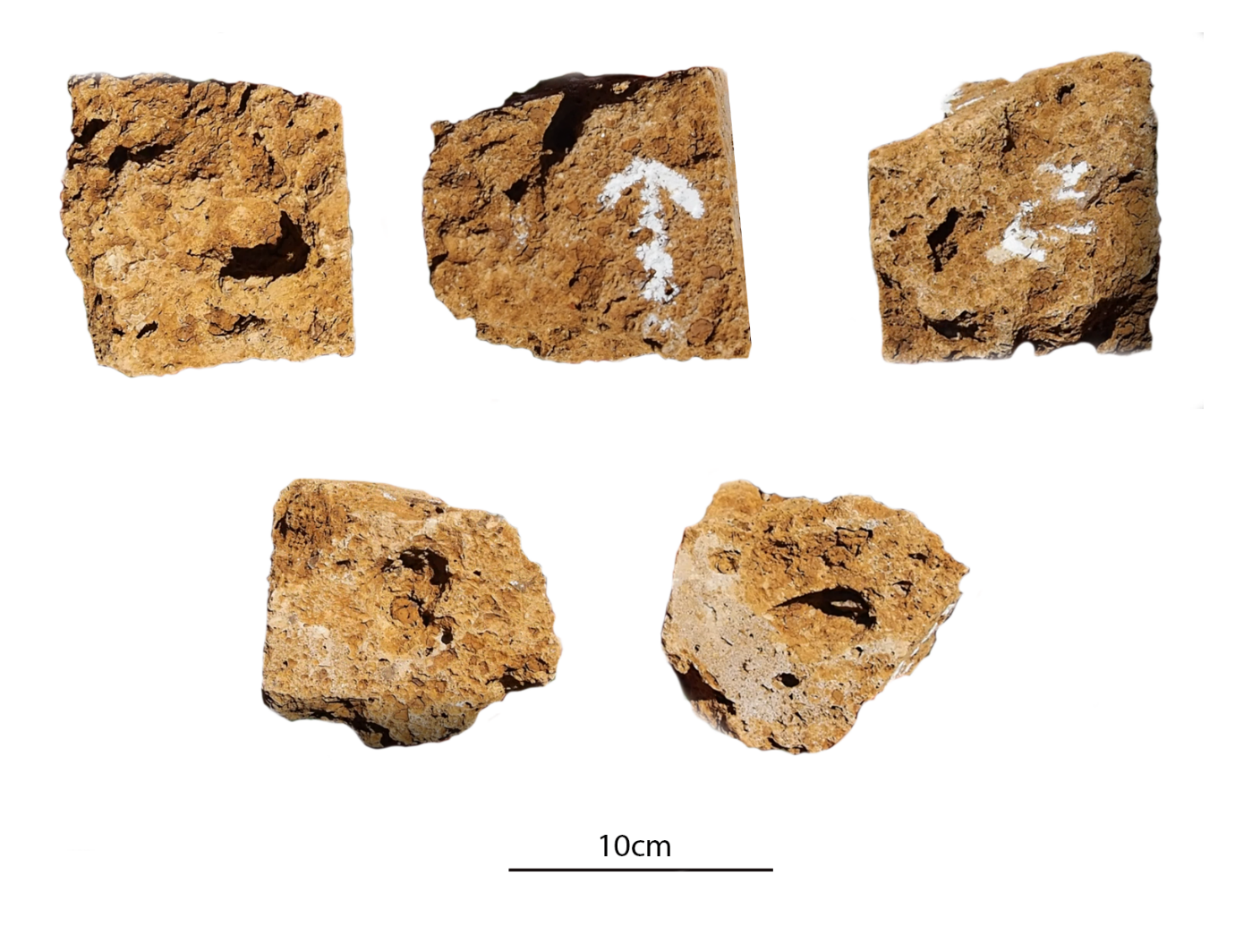


Figure S11. Reconsum18-48 block sample (top left) back facing radial plane (top middle) forward facing radial plane (top right) west facing transverse plane (bottom left) north facing tangential plane (bottom right) south facing tangential plane


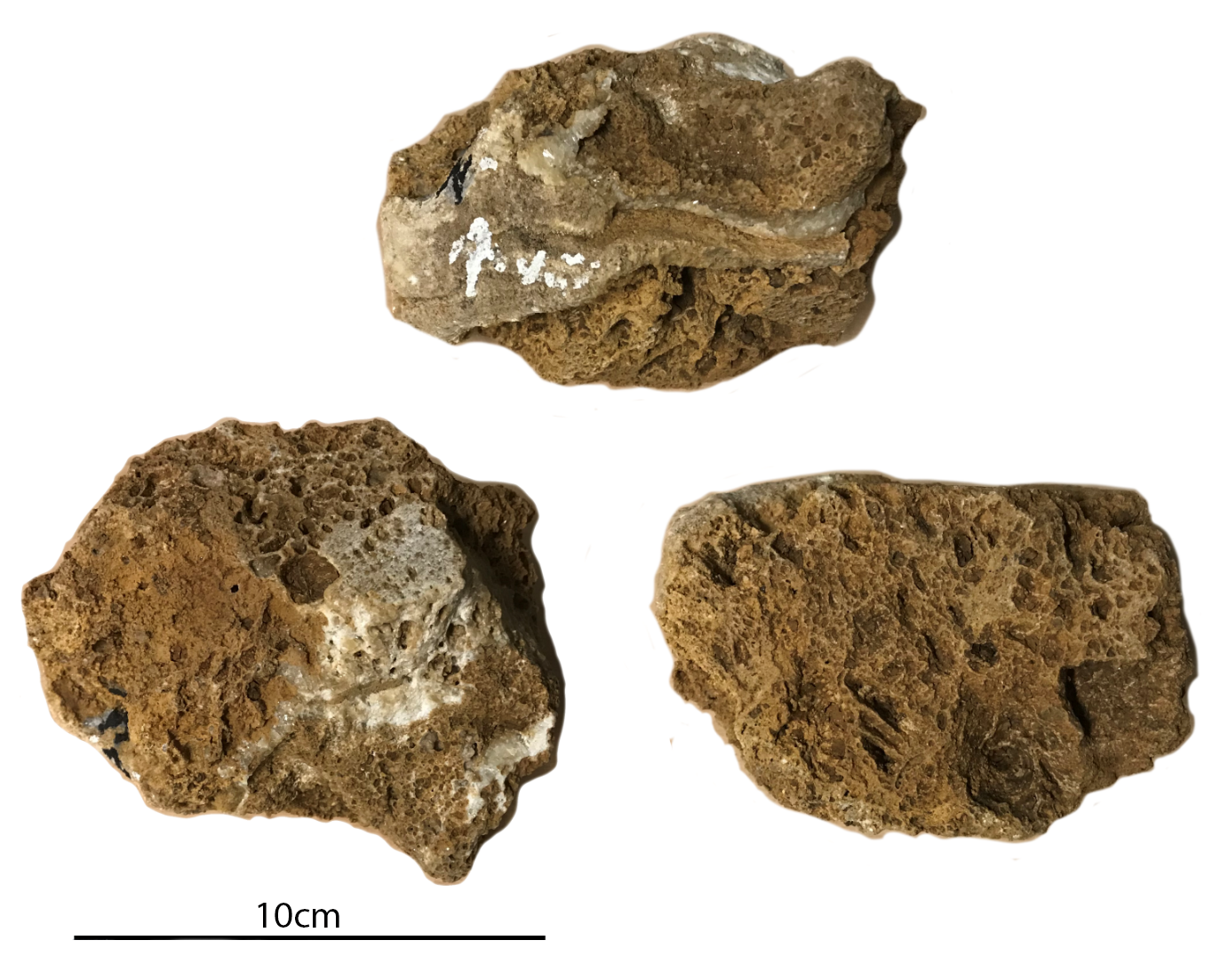


Figure S12. Reconsum18-72 block sample (top) forward facing radial plane (bottom left) upward facing transverse plane (bottom right) north facing tangential plane


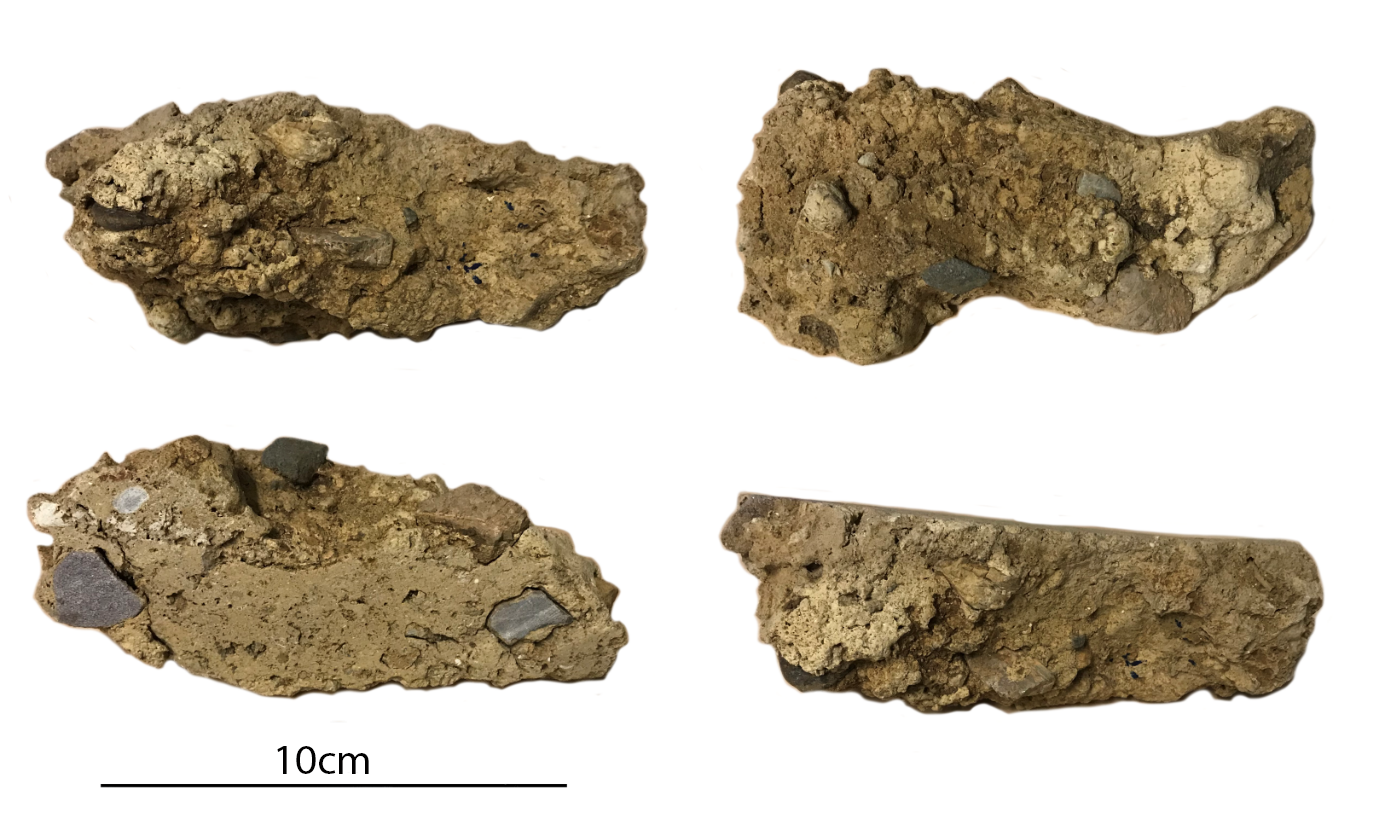


Figure S13. Reconsum18-51 block sample (top left) downward facing transverse plane (top right) forward facing south facing tangential plane (bottom left) backward facing north facing tangential plane (bottom right) upward facing transverse plane


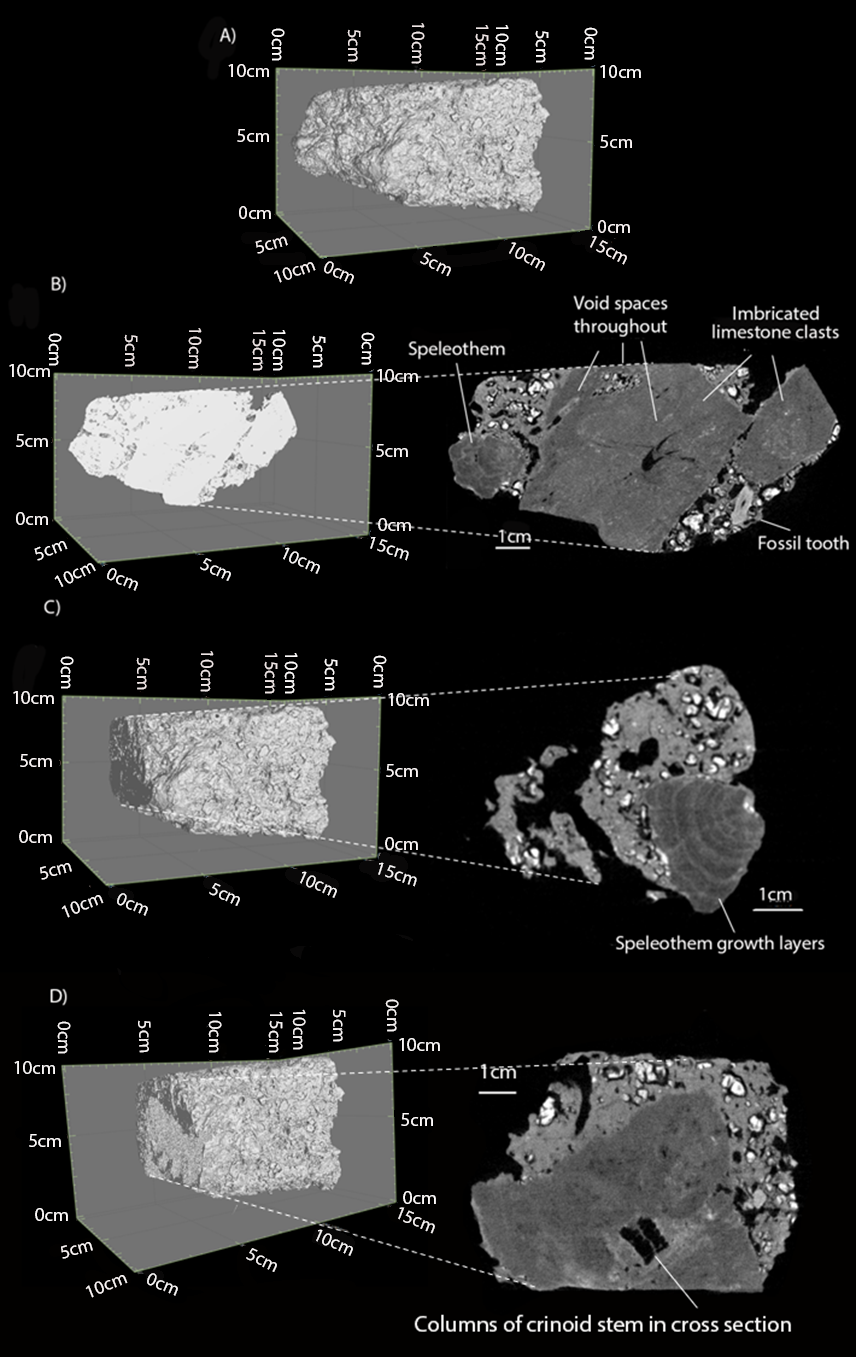


Figure S14. A) Three-dimensional tomographic reconstruction of breccia sample Reconsum18-52; B) Two-dimensional slice section through the central radial plane showing the carbonate mudstone matrix and the imbrication of the limestone clasts; C) Two-dimensional slice section through the tangential plane cut through the cross-section of the speleothem fragment in Figure 8B at 2 cm from the western periphery of the sample showing the diagnostic growth layers of the speleothem fragment; D) Two-dimensional slice section through the tangential plane at 6 cm from the western periphery of the sample showing the trace remnant of a crinoid fossil in the limestone clast.


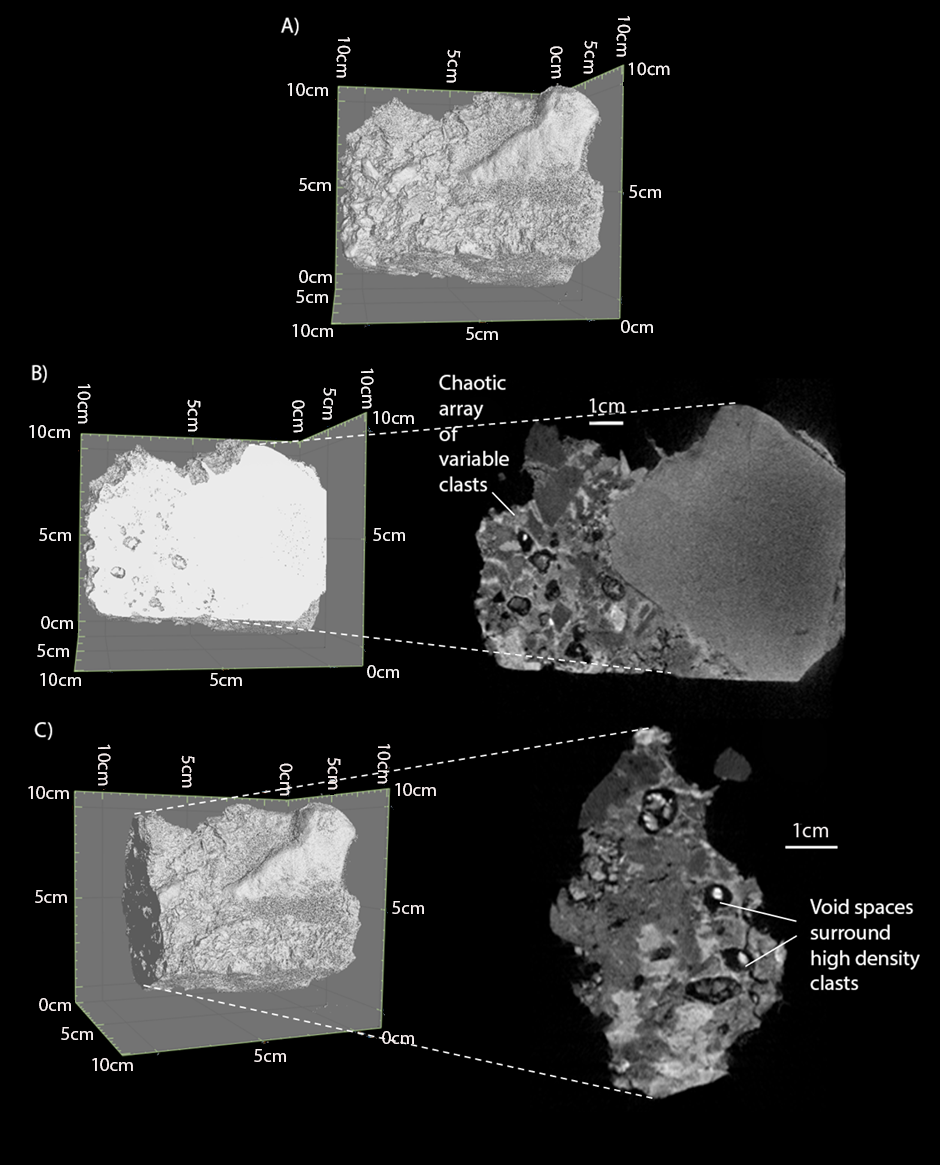


Figure S15. A) Three-dimensional tomographic reconstruction of breccia sample Reconsum18-50; B) Two-dimensional slice section through the central radial plane showing the variability in clast shape and neutron apparent density in the sample; C) Two-dimensional slice section through the tangential plane cut through the matrix 1.2 cm from the western periphery of the sample, showing the void spaces surrounding the internal clasts.


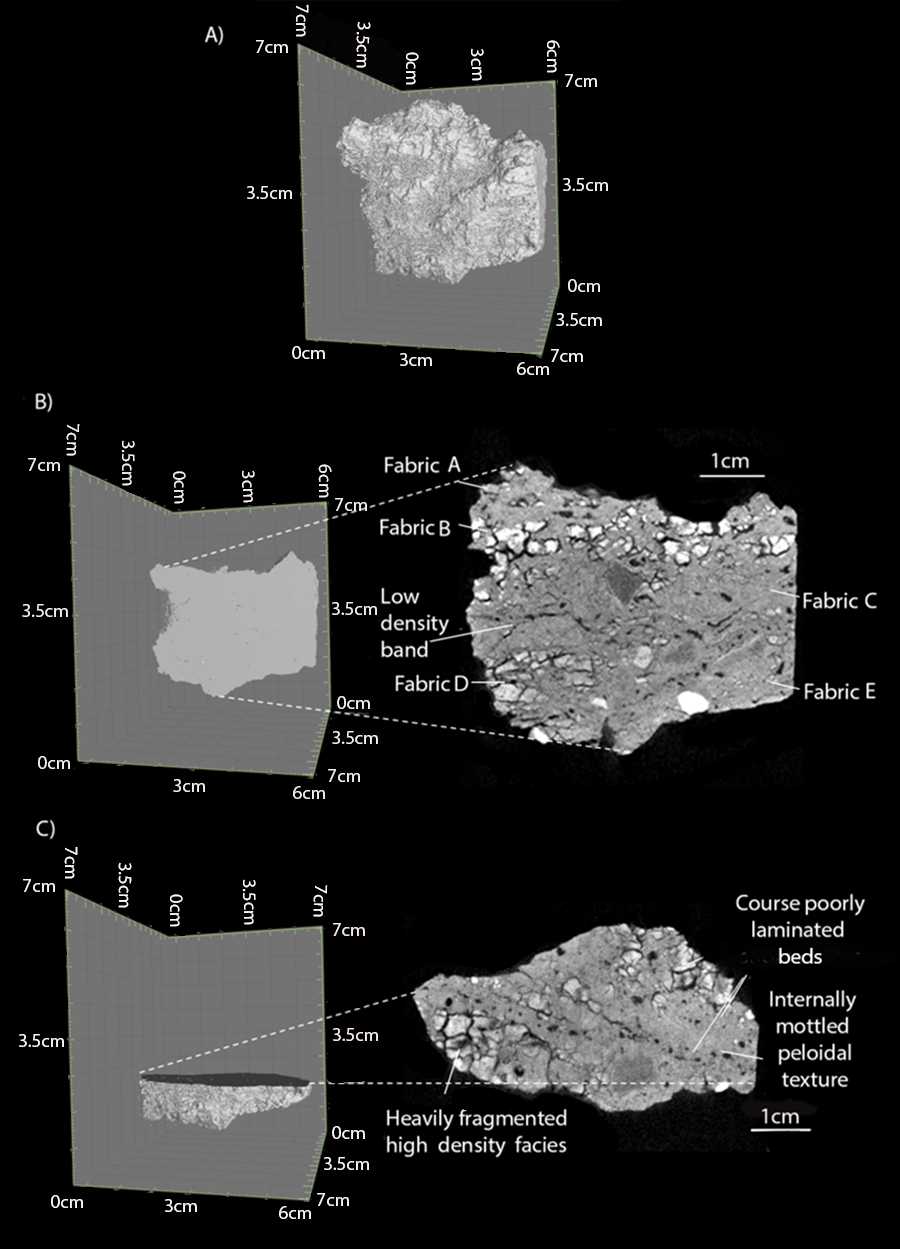


Figure S16. A) Three-dimensional tomographic reconstruction of breccia sample Reconsum18-47; B) Two-dimensional slice section through the central radial plane, evident is the bedding; C) Two-dimensional slice section through the transverse plane as a cross section of the laminations and void space evident in Fabric C and D in Figure 10B


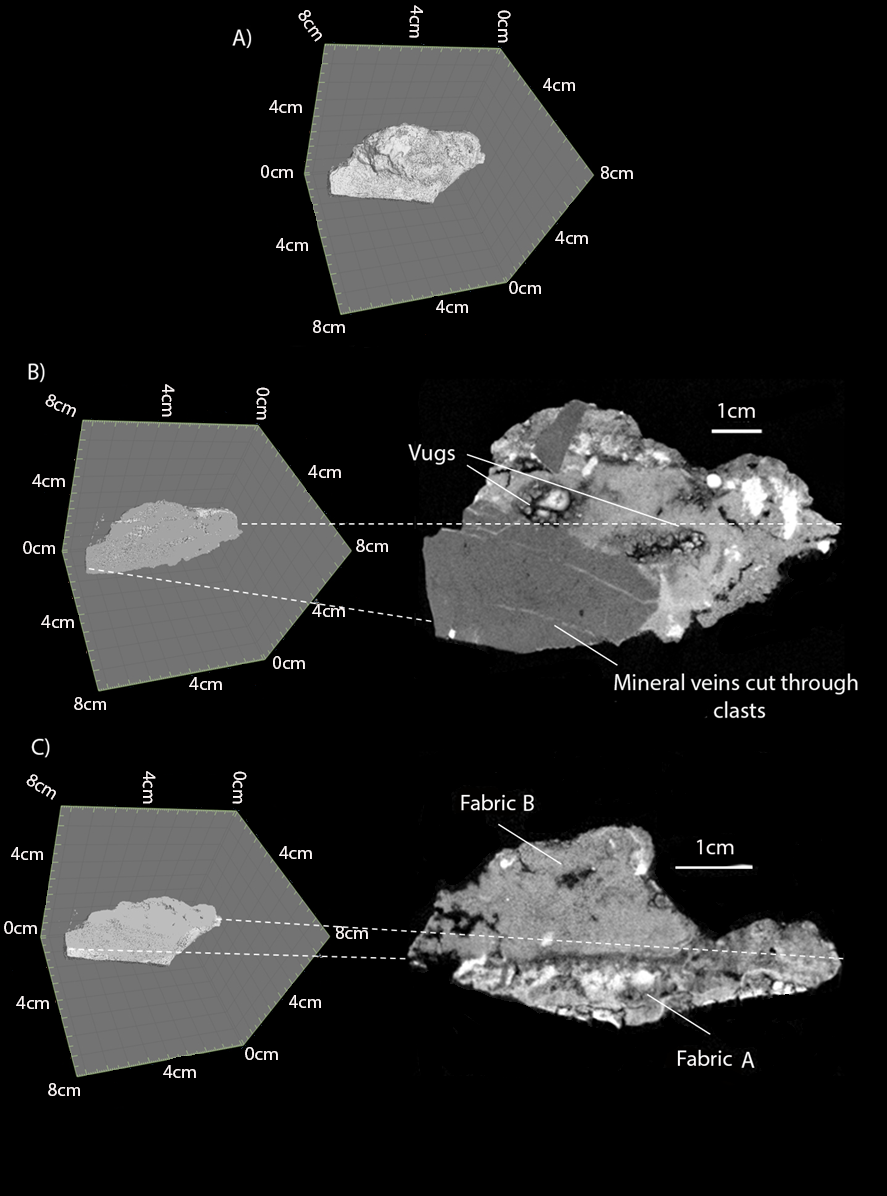


Figure S17. A) Three-dimensional tomographic reconstruction of breccia sample Reconsum18-46; B) Two-dimensional slice section through the central radial plane with evident vug cavities lined with precipitates; C) Two-dimensional slice section through the transverse plane at the discontinuity between Fabric A and B at 1.9cm from the downward facing periphery of the sample


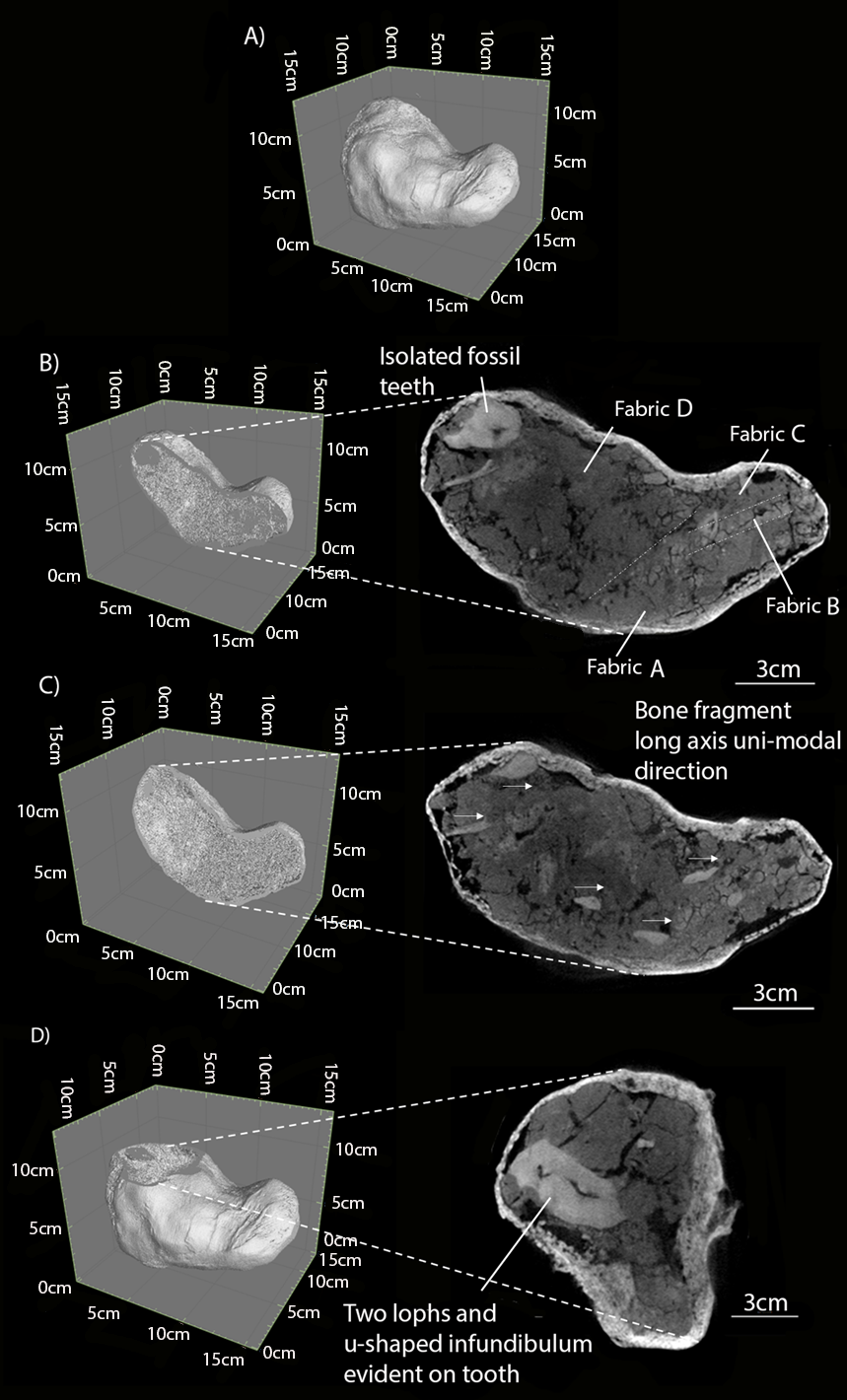


Figure S18. A) Three-dimensional tomographic reconstruction of breccia sample Reconsum18-73; B) Two-dimensional slice section through the central radial plane showing the individual fabrics. Dashed lines highlight the faint bedding; C) Two-dimensional slice section through the radial plane at 2.4 cm from the central slice towards the backward facing periphery of the sample showing the elongate bone fragments within; D) Two-dimensional slice section through the transverse plane cut through the cross section of the fossil tooth identified in Figure 12B at 1.5cm from the upward facing periphery of the sample showing the morphological detail of the large mammal tooth in fabric A. The thick white boundary surrounding the profile of the deposit in the scan images of this block sample is a supportive plaster cast used to prevent disintegration of the sample during NT scanning.


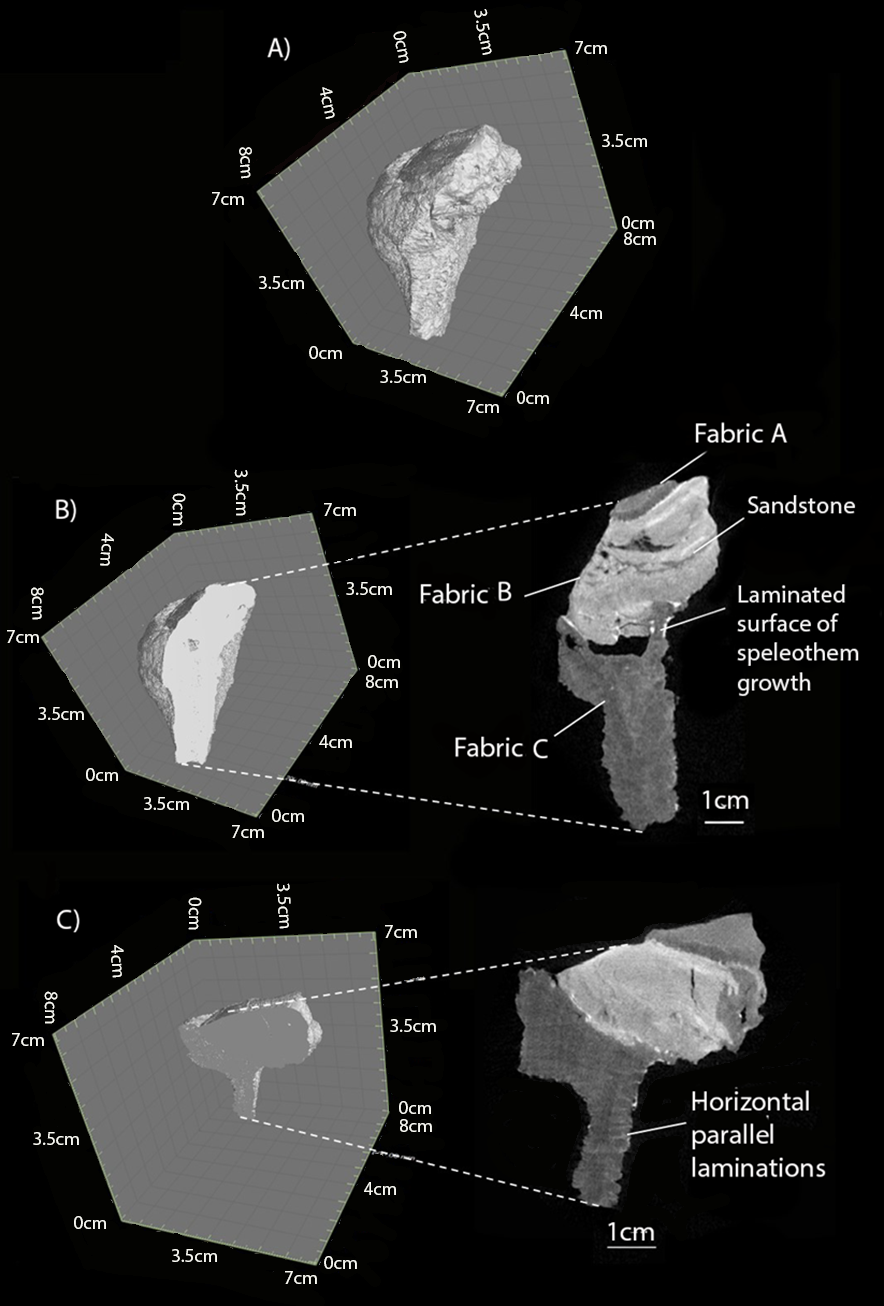


Figure S19. A) Three-dimensional tomographic reconstruction of breccia sample Reconsum18-55; B) Two-dimensional slice section through the central tangential plane showing a sandstone between two speleothem deposits; C) Two-dimensional slice section through the radial plane at 1.4 cm from the backward facing periphery of the sample showing the parallel laminations of speleothem growth


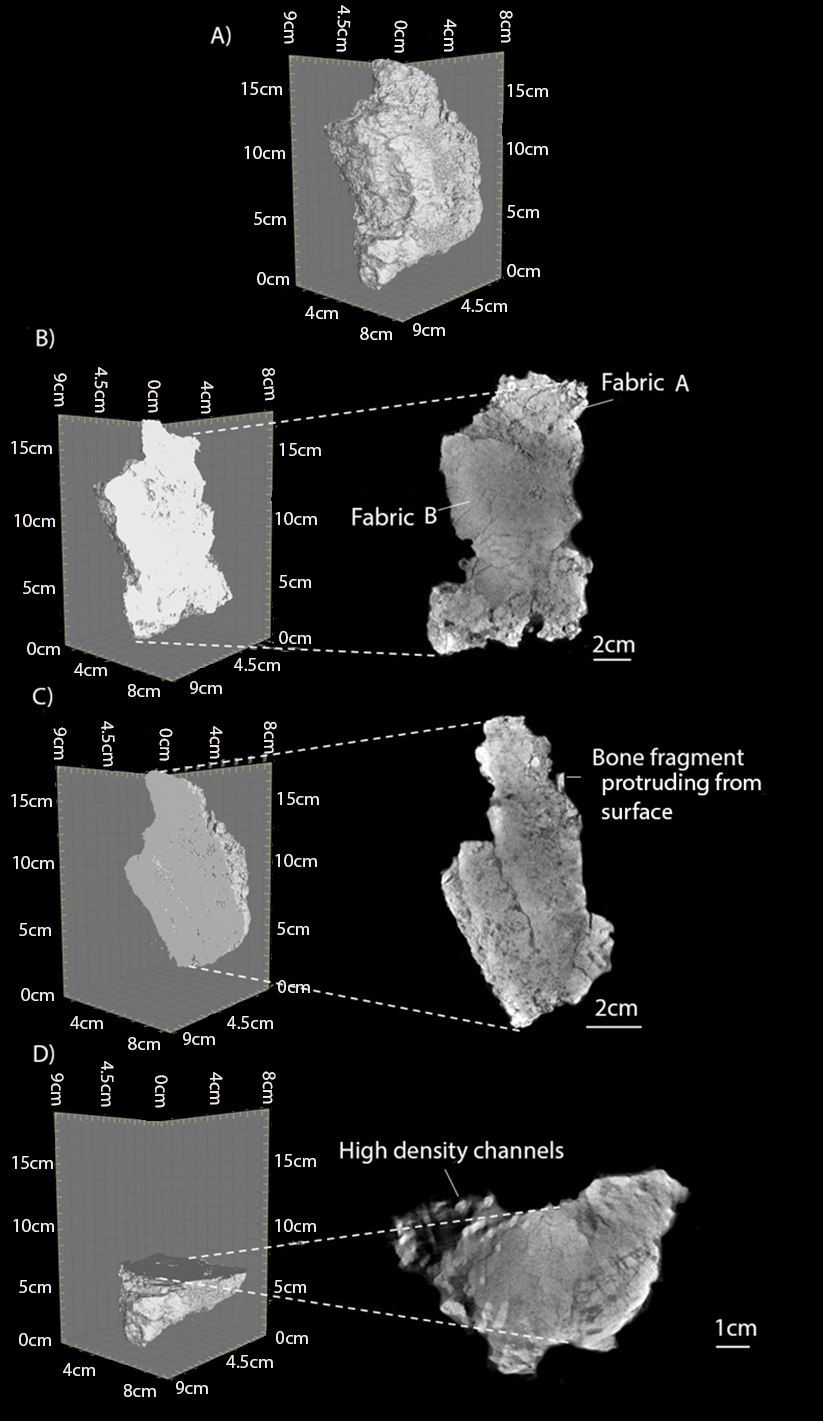


Figure S20. A) Three-dimensional tomographic reconstruction of breccia sample Reconsum18-49; B) Two-dimensional slice section through the central tangential plane showing the two mudstone fabrics; C) Two-dimensional slice section through the radial plane at 3.4 cm from the backward facing periphery of the sample showing the bone fragment protruding from the sample; D) Two-dimensional slice section through the transverse plane at 3.5 cm from the downward facing periphery of the sample showing the high density clasts in fabric A

*
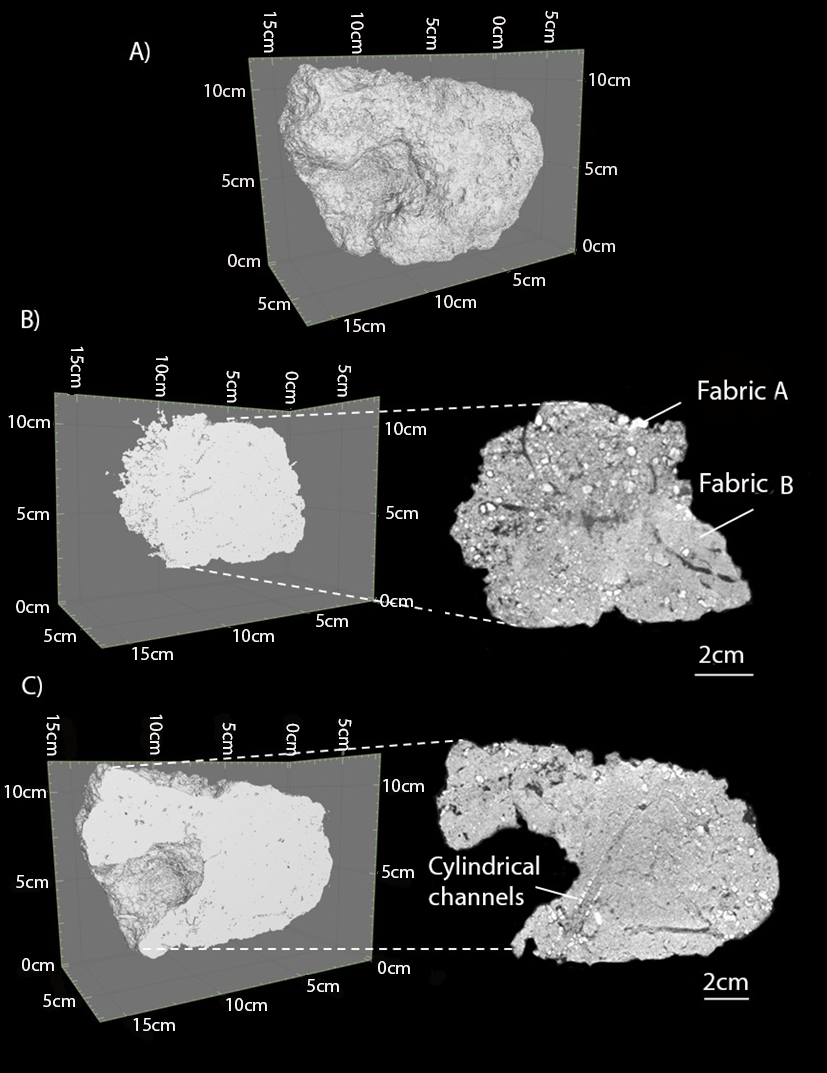
*

Figure S21. A) Three-dimensional tomographic reconstruction of breccia sample Reconsum18-71; B) Two-dimensional slice section through the radial plane showing clast supported fabric A and matrix supported fabric B; C) Two-dimensional slice section through the central tangential plane at 2.4 cm from the eastern periphery of the sample showing the plant root trace fossils lined by fractured calcite deposits

*
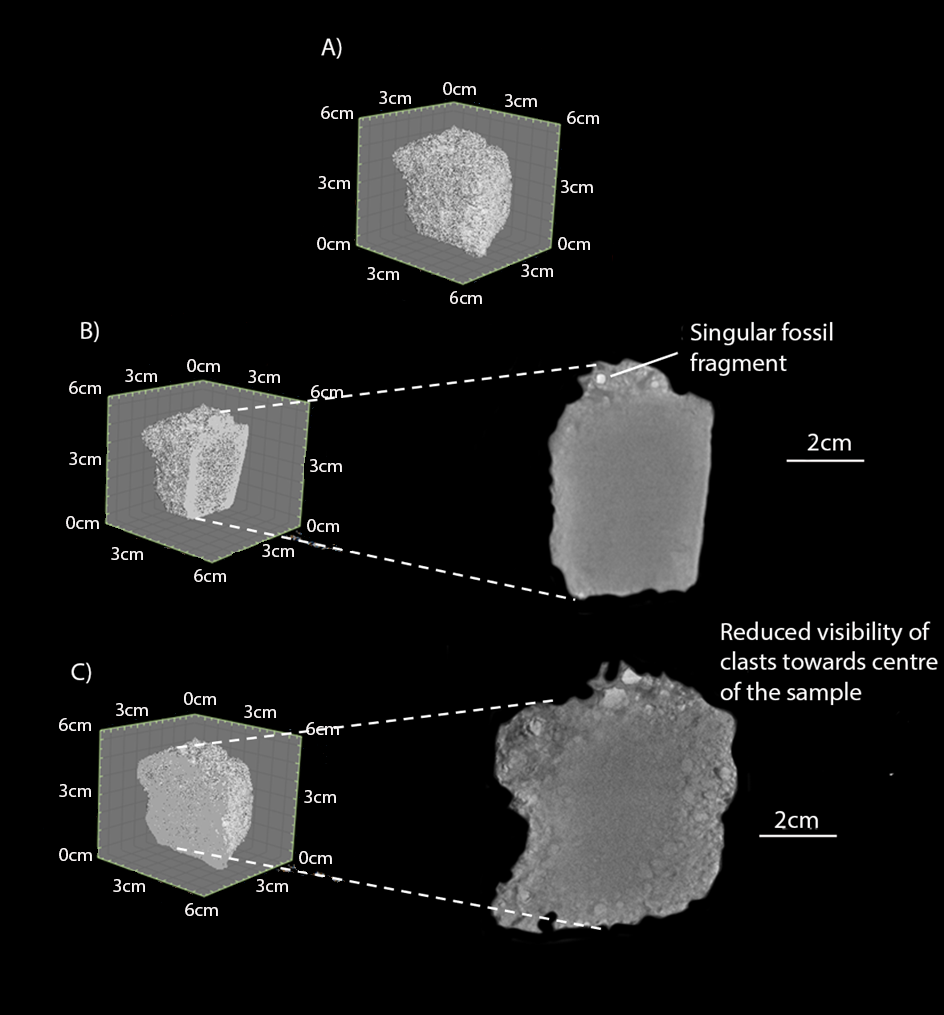
*

Figure S22. A) Three-dimensional tomographic reconstruction of breccia sample Reconsum18-54; B) Two-dimensional slice section through the central radial plane showing the rounded fossil fragment; C) Two-dimensional slice section through the central tangential plane showing the clast-supported sandstone fabric of the sample.

*
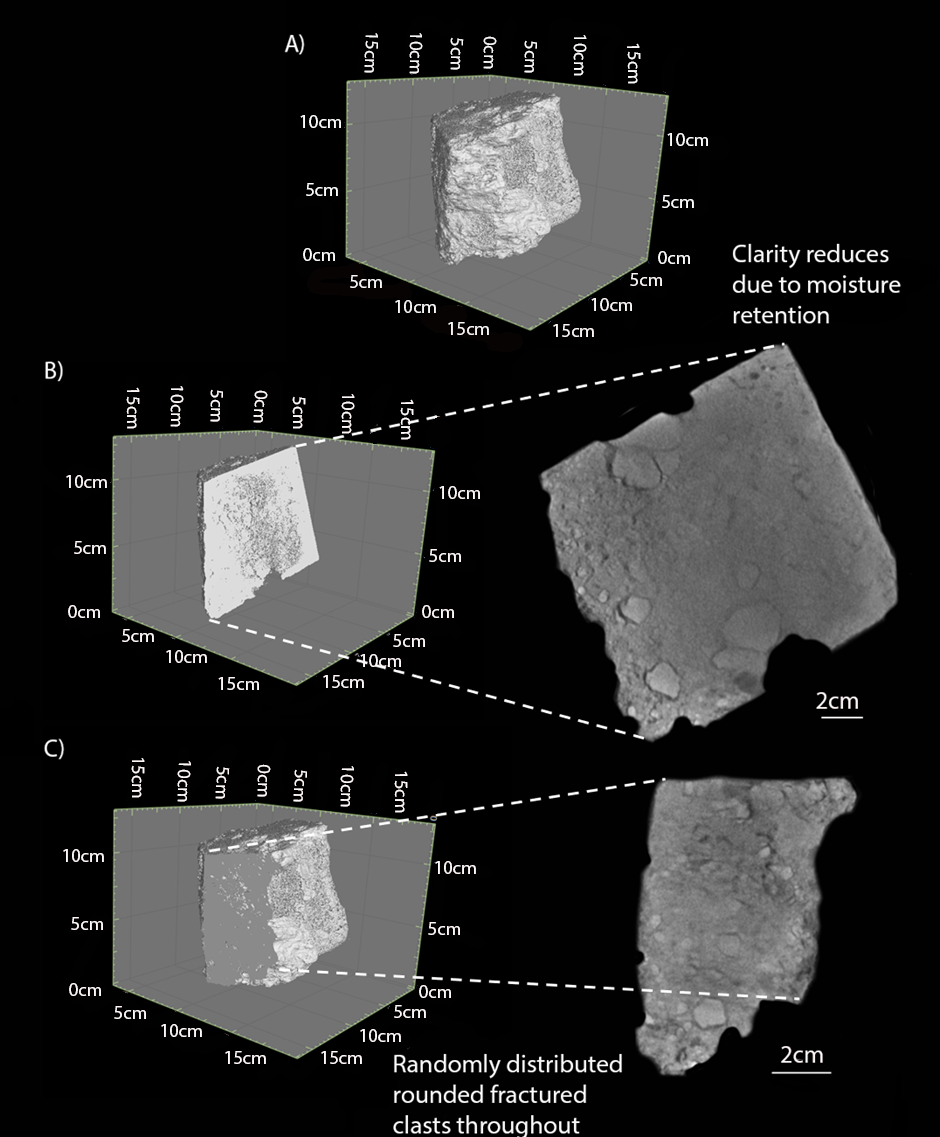
*

Figure S23. A) Three-dimensional tomographic reconstruction of breccia sample Reconsum18-48; B) Two-dimensional slice section through the radial plane showing the fractured clasts surrounded by void space floating in the matrix; C) Two-dimensional slice section through the tangential plane showing the variation in density of the internal clasts


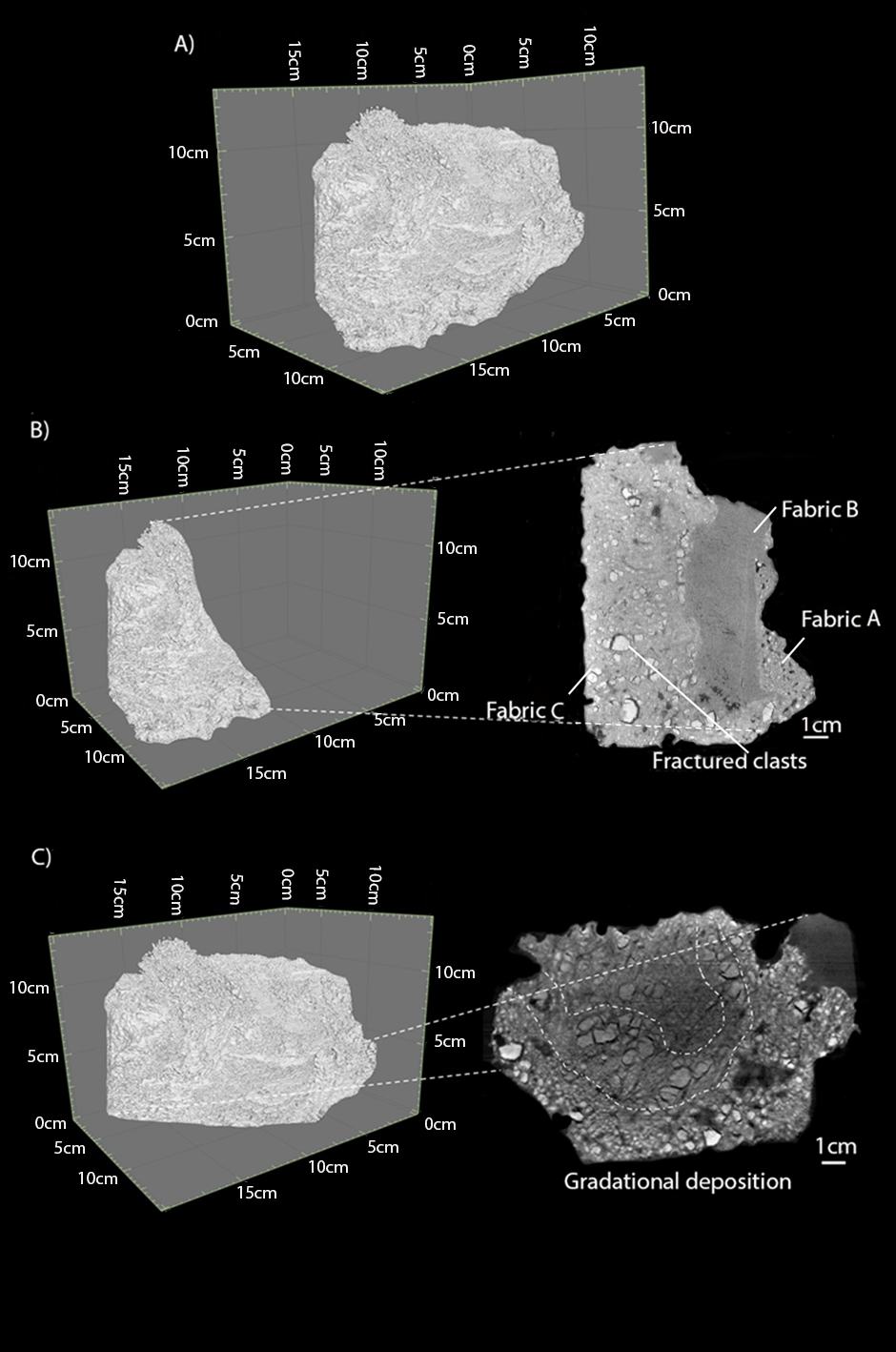


Figure S24. A) Three-dimensional tomographic reconstruction of breccia sample Reconsum18-72; B) Two-dimensional slice section through the tangential plane at 5.4 cm from the western periphery of the sample showing the two sandstones surrounding a speleothem deposit; C) Two-dimensional slice section through the transverse plane cut through the fractured clast material of Fabric C in Figure 18B at 5.1 cm from the downward facing periphery of the sample showing the increasing fracturing and disintegration of clasts towards the sample periphery


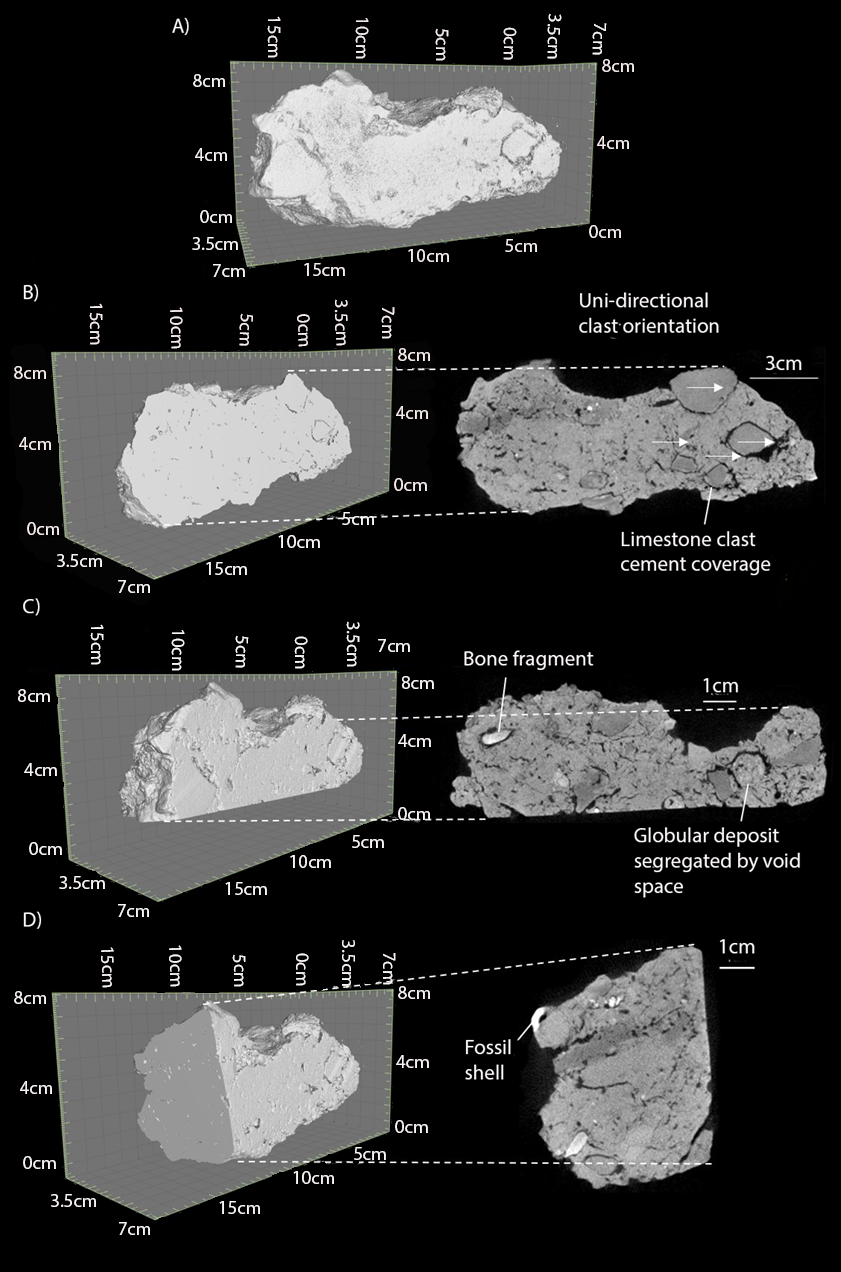


Figure S25. A) Three-dimensional tomographic reconstruction of breccia sample Reconsum18-51; B) Two-dimensional slice section through the central radial plane showing the uni-directional orientation of the internal clasts; C) Two-dimensional slice section through the radial plane at 3.2 cm from the north facing periphery of the sample showing the soil glaebules in the sandstone matrix; D) Two-dimensional slice section through the tangential plane at 1.8 cm from the western periphery of the sample showing a fossil bone fragment and a gastropod shell
